# Abstracts of the 2025 51st Annual NATAS Conference

**DOI:** 10.3390/polym17162196

**Published:** 2025-08-11

**Authors:** Kenneth L. Kearns, Camille Bishop, Lawrence Judovits, John Rosener, Cathy Stewart, Tina Adams

**Affiliations:** 1 Dow Chemical Company, Midland, MI 48674, USA; 2 Chemical Engineering and Material Science, Wayne State University, Detroit, MI 48201, USA; 3 Independent Researcher, Bluffton, SC 29909, USA; 4 Lawrence Livermore National Laboratory, Livermore, CA 94550, USA; 5 Intertape Polymer Group, Marysville, MI 48040, USA; 6 The Lubrizol Corporation, Wickliffe, OH 44092, USA

**Keywords:** thermal analysis, sustainable polymers, thermophysical properties, batteries, kinetics, rheology, composites

## Abstract

The North American Thermal Analysis Society (NATAS) is pleased to announce its 51st Annual Conference, held jointly with the IX International Baekeland Symposium. This premier event unites scientists, practitioners, and students from academia, industry, and government to explore the forefront of materials science. The NATAS conference provides a dynamic forum for attendees to delve into the latest advancements in thermal analysis, rheology, and materials characterization. The technical program will highlight new developments in instrumentation and software, alongside practical applications across a wide range of industries. Concurrently, the Baekeland Symposium will showcase cutting-edge scientific, technical, and industrial innovations in the field of high-performance thermosetting polymers. The synergy of this joint meeting creates a unique platform for cross-disciplinary collaboration, fostering the exchange of novel ideas and sparking new research opportunities. Featuring technical presentations, poster sessions, and plenary lectures from renowned experts and emerging graduate students, the conference offers an ideal environment for networking and professional development. We invite you to join us to discover state-of-the-art techniques, discuss groundbreaking research, and connect with peers and leaders in the thermal and materials community.

## 1. Plenary Lectures

### 1.1. New Simulation and Modeling Tools to Understand Rheology, Processing, and Crystallization of Polyethylene Melts

Ronald G. Larson *
University of Michigan
* Correspondence: rlarson@umich.edu

**Abstract:** New simulation methods are contributing to solving an old problem: how molecular weight and branching distribution, and rheology and processing conditions control properties such as modulus and toughness of polyethylene film. In one part of this talk, a practical method is presented that accounts for the effects of short- and long-chain branching on rheological properties of commercial polyethylene through the use of an “optimal ensemble” of chains, which can be used to predict chain orientation in a processing flow such as film blowing. In a second part, molecular dynamics simulations are used to show that both primary and secondary nucleation proceed through a nematic-like intermediate. We show that short-chain branches are partially expelled from the growing crystal to an extent dependent on branch length, and by measuring the degree of expulsion, we can infer the “defect energy” induced by trapping of branches of various lengths in the crystal. We develop a “stem-by-stem” model for the effect of short-chain branching on the rate of crystallization of polyethylene that is in accord with experiments, and can be useful in modeling crystallization during film blowing.

### 1.2. Viscoelasticity and Light-Matter Interactions at GHz Frequencies

John Kieffer *
University of Michigan
* Correspondence: kieffer@umich.edu

**Abstract:** Brillouin light scattering (BLS) is a non-invasive technique that probes the propagation velocity and attenuation of thermal phonons that exist in thermodynamic equilibrium within any condensed matter phase at finite temperatures. The elastic storage modulus is derived from the propagation velocity, whereas the attenuation coefficient yields the loss modulus. The measurement is performed without mechanical contact with the sample. Therefore, it does not disrupt the equilibrium state of the material under investigation and requires only micron-sized sample sizes. The technique can be applied to characterize bulk materials, thin films, or single cells. This presentation illustrates the capabilities of BLS and the type of information revealed by these measurements. We review two case studies: (i) The unique experimental setup in our lab combines a miniature tensile tester placed into the BLS optical path to probe the elastic properties of materials while uniaxially strained. BLS yields the adiabatic modulus, while the isothermal modulus is derived from the measured stress-strain curves. Upon straining, the elastic moduli of polymers drop precipitously, but during stress relaxation at constant strain, both the isothermal and adiabatic moduli reconstitute, tending towards the unstrained values. While the former is implicit in the Maxwell-Wiechert (MW) model, the latter is not. Indeed, the structure rearranges to optimize molecular packing and maximize the non-bonding interactions. Hence, viscoelastic property changes require descriptions beyond simple MW models. (ii) The adiabatic loss modulus of a melt can be related to its viscosity. The temperature dependence of viscosity is at the heart of glass formation and reflects the structural developments that occur in the supercooled liquid as it passes through the glass transition regime. We demonstrate that the high-frequency complex mechanical modulus determined using Brillouin light scattering yields the same values for the viscosity as a function of temperature as zero-frequency rotating cylinder viscometry when interpreted using a modified MW model, which is based on the assumption of a correlative activation free energy (CAFE) for the viscous dissipation process. Accordingly, both the activation energy and entropy vary with temperature, which implies that the free energy landscape topography evolves along with the molecular structure as the material transitions from a liquid to a glass.1 Conversely, we can determine the activation energy and entropy by fitting viscosity data using our CAFE model. We have analyzed the viscosity data for 849 glass compositions, confirming the robustness of our model and yielding results that enable deeper insights into structural relaxation mechanisms. We elaborate on the relationship between the rate of change of the energy landscape and fragility, the importance of activation entropy, and the influence of materials chemistry. (Acknowledgement: NSF-DMR 1610742.)

**References**

1. Kieffer, J. *‘Brillouin Light Scattering,’ in Modern Glass Characterization*; Affatigato, M., Ed.; Wiley & Sons: Hoboken, NJ, USA, 2015; p. 107.
2. Beg, M.C.; Kieffer, J. Fragility and the rate of change of the energy landscape topography. *J. Non-Cryst. Solids X* **2022**, *14*, 100101.
3. Beg, M.C.; Byeon, J.; Berman, N.; Kieffer, J. Correlative Activation Free Energy (CAFE) Model: Application to Viscous Processes in Inorganic Oxide Glass-Formers. *Acta Mater.* **2025**, *283*, 120538.

### 1.3. NATAS Award for Outstanding Achievement: Differential Scanning Calorimetry (DSC) of Polymers: Tips and Tricks (Or Hacks for the Younger Crowd)

Brian Grady *
University of Oklahoma
* Correspondence: bpgrady@ou.edu

**Abstract:** This talk will be to highlight some 35 years of learning in performing DSC experiments on polymers, interspersed with personal recollections. Four items will be discussed.

Over the last 35 years, perhaps the most underreported improvement in changes in DSC experiments has been the improvement in baseline stability. In particular, good baseline stability is a requirement for accurate heat capacity measurement. With a high-quality DSC, the limiting situation has been reached in the case of good sample/pan contact, namely that the specifics of how the sample sits in the pan limit the error in the measurement.

One reason to measure heat capacity is that a mass balance can determine the amount of crystalline, amorphous, and rigid amorphous fraction (RAF). In this section, the RAF will be explained, and some studies on ionomers and composites will be discussed.

The importance of sample thickness with respect to heating or cooling rate will be discussed. This problem becomes much harder to quantify in cases where crystallization or melting occurs, since there is a local intake or release of heat. Our studies in this area will be highlighted.

DSC allows for the measurement of thermal conductivity for samples with low thermal conductivity. Our work in this area will be highlighted, and the limits of the technique will be discussed.

### 1.4. NATAS Fellowship Award: Combined Influence of Pressure and Shear Flow on the Crystallization of Isotactic Polypropylene

Alicyn Rhoades *, Benson Jacob, Jörg Läuger, Xiaoshi Zhang, Ralph Colby, Markus Nemeth and Kirt Page
Penn State—Behrend
* Correspondence: amh234@psu.edu

**Abstract:** In many melt processing techniques, polymers undergo rapid melting and deformation under pressure, followed by a rapid cooling or quench to solidification—conditions far from equilibrium and difficult to explore using traditional rheology instrumentation. To better understand polymer flow and crystallization under pressure, we studied a commercial isotactic polypropylene using new rheological tools capable of applying simultaneous rotational shear flow and pressure. As expected, the combination of flow and pressure accelerated crystallization kinetics. However, the most striking finding was the profound impact that pressure seems to have on the crystallization mechanism and resulting morphology.

At moderate pressures (~2 bar), typical shish kebab morphologies resulted after shear flow. However, at elevated pressures of 100–180 bar, alignment was suppressed, and morphologies were more isotropic despite shear flow. This shift may suggest a transition to a different crystallization pathway that is not simply accelerated but fundamentally altered by pressure. Unexpectedly, crystallization occurred above nominal melting conditions, indicating a pressure-induced suppression of chain mobility. X-ray scattering revealed the presence of a new crystalline peak exclusive to samples produced under the shear/pressure combination, potentially indicating a pressure-induced polymorphism. These morphologies resulted in melting temperatures that were up to 10 K higher than their quiescently crystallized counterparts. Together, these results point to pressure-driven processes that influence not only crystallization kinetics and resulting morphology, but to enhanced bulk properties that are potentially important for industrial adoption.

## 2. Session: Batteries

### 2.1. Lignin-Based Thermally Stable Separators for Lithium-Ion Batteries

Chengcheng Fang *
Michigan State University
* Correspondence: cfang@msu.edu

**Abstract:** Separators are critical components in lithium-ion batteries (LIBs), preventing internal short circuits, mitigating thermal runaway, and influencing rate capability and cycling performance. However, current polyolefin separators suffer from limitations, such as high thermal shrinkage, relatively poor wettability, and inadequate long-term stability, impacting safety and cycle life in critical applications like electric vehicles. Here, a single-layer lignin-based ultrathin separator (as thin as 15 µm) with exceptional intrinsic thermal stability and cycling performance is demonstrated. The separator is fabricated using lignosulfonate, a natural polymer derived as a byproduct of chemical pulping and biorefinery processes. By employing a dry fibrillation method, the process achieves low energy consumption and a 100% raw material conversion rate, highlighting its scalability and sustainability. Interfacial studies reveal that the improved cycling performance in both graphite||NMC811 and Si-Gr||NMC811 cells is attributed to the abundant sulfonate functional groups in lignosulfonates, which promote the formation of a sulfur-rich cathode/solid electrolyte interphases (CEI/SEI) with low resistance in both the cathode and anode. The high thermal stability, manufacturing feasibility, battery performance, and low cost of such lignin-based separators offer new inspiration for developing next-generation, single-layer functional separators tailored for high-performance LIBs.

**Reference**

1. Jia, H.; Liu, J.; Liu, B.; Kuphal, R.; Mottini, V.; Monday, P.; Ball, M.; Li, J.; Nejad, M.; Fang, C. Lignin-Based Separators for Lithium-Ion Batteries via a Dry Fibrillation Method. *Adv. Mater.* **2025**, *37*, 2419694

### 2.2. Oxidation Sensitivity and Thermal Decomposition of Halide-Doped Argyrodite Solid Electrolytes via TGA/DSC and Gas Analysis Method

Hernando Gonzalez Malabet *
General Motors
* Correspondence: hernandojesus.gonzalezmalabet@gm.com

**Abstract:** Sulfur-based solid electrolytes are central to the development of safer, high-performance lithium-ion batteries. Among these, halide-doped argyrodite materials such as Li_6_PS_5_Cl and Li_6_PS_5_Br have attracted significant attention due to their high ionic conductivities and enhanced moisture stability [1,2]. However, their thermal reactivity and potential flammability—particularly under oxidative conditions—remain underexplored and are not well understood within the scientific community [3,4].

This study investigates the oxidation sensitivity of three argyrodite solid electrolytes—Li_5.5_PS_4.5_Cl_1.5_, Li_6_PS_5_Cl, and Li_6_PS_5_Cl_0.5_Br_0.5_—using simultaneous thermogravimetric analysis (TGA) and differential scanning calorimetry (DSC) under dry air and argon atmospheres, with inert alumina crucibles. These thermal analyses are complemented by semi-in situ sulfur dioxide (SO_2_) gas detection using gas chromatography–mass spectrometry (GC/MS), enabling the identification of sulfur release and assessment of flammability risks at elevated temperatures. The results reveal distinct decomposition profiles and SO_2_ evolution behaviors, which are correlated with the degree of halide mixing and molar disorder in the crystal structure.

Our findings aim to clarify whether halide substitution or molar disorder plays a more significant role in mitigating oxidation-driven decomposition by stabilizing the anionic framework and reducing sulfur volatility. This work not only provides critical insights into material design strategies for suppressing thermal reactivity but also introduces a novel methodology for evaluating the thermal safety of solid electrolytes in next-generation battery systems.

**References**

1. Wang, Q.; Zhou, Y.; Wang, X.; Guo, H.; Gong, S.; Yao, Z.; Wu, F.; Wang, J.; Ganapathy, S.; Bai, X.; et al. Designing lithium halide solid electrolytes. *Nat. Commun.* **2024**, *15*, 1050. https://doi.org/10.1038/s41467-024-45258-3.
2. Wang, D.; Shi, H.; Wang, S.; Wu, X.; Jiang, W.; Liang, S.; Xu, Z. New insights into Li-argyrodite solid-state electrolytes based on doping strategies. *Coord. Chem. Rev.* **2024**, *508*, 215776. https://doi.org/10.1016/j.ccr.2024.215776.
3. Guo, L.; Zheng, J.; Zhao, L.; Yao, Y. Interfacial instabilities in halide-based solid-state batteries. *MRS Bull.* **2023**, *48*, 1256–2023. https://doi.org/10.1557/s43577-023-00607-3.
4. Yersak, T.A.; Malabet, H.J.G.; Yadav, V.; Pieczonka, N.P.W.; Collin, W.; Cai, M. Flammability of sulfide solid-state electrolytes β-Li3PS4 and Li6PS5Cl: Volatilization and autoignition of sulfur vapor—New insight into all-solid-state battery thermal runaway. *J. Energy Chem.* **2025**, *102*, 651–660. https://doi.org/10.1016/j.jechem.2024.11.031.

### 2.3. From Winter to Summer: How Seasonal Climate Variation Shapes Battery Lifespan and Mileage

Hanyu Bai *, Qiuhao Hu, Yao Ren, Weiran Jiang and Ziyou Song
University of Michigan
* Correspondence: hanyubai@umich.edu

**Abstract:** Understanding how temperature fluctuations affect lithium-ion batteries in electric vehicles (EVs) is essential for optimizing their performance, ensuring safety, and extending their operational lifespan. While the short-term effects of temperature on battery performance and the influence of constant thermal conditions on overall lifespan are well documented, the long-term impact of seasonal temperature fluctuations remains largely underexplored. This work investigates how ambient temperature fluctuations affect battery degradation trajectories and real-world mileage. We analyzed the impact of cumulative thermal exposure on critical battery health metrics. Our findings reveal that batteries exposed to broader seasonal extremes—particularly high summer temperatures and low winter temperatures—exhibit accelerated degradation and reduced mileage over time. We also discuss the limitations of traditional calendar and cycle life models in capturing these effects and highlight the need for temperature-aware lifetime prediction frameworks. These insights underscore the importance of seasonally adaptive lifecycle management strategies to ensure battery reliability and performance across diverse climates.

### 2.4. Acoustically Driven Thermal Management of Battery Packs in Electric Vehicles

Young Jin Lee *, Mikko Hendricks and Solomon Adera
University of Michigan
* Correspondence: xerojin@umich.edu

**Abstract:** As lithium-ion batteries in electric vehicles are pushed to deliver higher energy density and faster charge–discharge rates, effective thermal management becomes increasingly critical. A major challenge lies in suppressing transient thermal hotspots within the battery pack, which can accelerate aging, degrade performance, and/or even trigger the catastrophic events of thermal runaway. While conventional approaches such as cold plates and liquid-cooled jackets are widely adopted in practice, they often lack the spatial and temporal resolution to address highly localized and rapidly changing cyclic heat loads. Moreover, passive systems are inherently limited by their static nature and delayed thermal response, underscoring the need for active and tunable cooling strategies. In this study, we propose a method for localized thermal regulation based on acoustically enhanced boiling, where discrete vapor bubbles form at nucleation sites on a heated surface and detach into the surrounding liquid. The system employs high-frequency (1.7 MHz) ultrasonic waves applied to a pool boiling surface, in which the heated surface is submerged in a quiescent liquid (ethanol in this case). By adjusting the acoustic input voltage, we modulate bubble departure dynamics to enhance the heat dissipation rate. Moderate acoustic forcing (1 V) increases the critical heat flux (CHF)—the maximum heating power per unit area that can be removed without triggering boiling crisis—by 42%, while also improving the heat transfer coefficient (HTC), defined as the heat flux per degree of wall superheat (excess temperature above saturation). High-speed imaging confirms that acoustic forcing increases bubble departure frequency, reduces bubble departure diameter, and sustains microlayer rewetting, which is the replenishment of the thin liquid film beneath a growing bubble—a critical mechanism for maintaining high local heat transfer rate. Excessive forcing (6 V), however, destabilizes the microlayer and promotes bubble coalescence, thereby reducing HTC. These results establish acoustic actuation as a compact, non-invasive, and electrically tunable approach for spatially resolved thermal management. With further engineering, this technique could be integrated into battery cooling plates and/or thermal interface layers to dynamically respond to the transient heat generation in battery packs. Our experimental findings promise a viable pathway towards active thermal control of next-generation electric vehicle battery packs.

### 2.5. Wide Band Gap Semiconductor Thermal Management Using Direct Immersion Cooling

Biruk Teka Gidreta *, Runshuang Guo and Solomon Adera
University of Michigan
* Correspondence: birukg@umich.edu

**Abstract:** The increasing power density in wide band gap (WBG) semiconductors such as Gallium Nitride (GaN) and Silicon Carbide (SiC), which enable vehicle electrification, presents severe thermal management challenges. The local heat generation in these devices can exceed 1 kW/cm^2^, which can exceed the heat dissipation capacity of traditional cooling solutions such as forced air convection and/or liquid cooling. Recently, there has been a sustained effort in using immersion cooling as a viable thermal management solution for WBG semiconductors. Immersion cooling of heat-dissipating surfaces eliminates the high solid-solid interface resistance and takes advantage of the highly efficient phase change processes. In this study, we evaluate the thermal performance of immersion cooling using a dielectric fluid on a GaN-based field-effect transistor (FET) mounted on a custom PCB. We compare the heat transfer coefficient (HTC) achieved by immersion cooling with that obtained using an extruded aluminum heat sink. Furthermore, we validate our experimental results using simulations on ANSYS Icepak. The results provide a quantitative basis for immersion cooling performance and can help guide the package design and dielectric fluid selection for immersion cooling of WBG semiconductors that play critical roles in the power electronics that manage how batteries are charged, discharged, and used.

### 2.6. Temperature Estimation of Pouch Cell with Indirect Liquid Cooling Based on Physics-Informed Machine Learning

Zheng Liu *
University of Michigan-Dearborn
* Correspondence: zhengtl@umich.edu

**Abstract:** With the development of the indirect liquid cooling system for the pouch cell in energy storage systems, temperature estimation becomes an important factor in design and operation. Traditional methods rely on experiments and finite element simulations, which are time-consuming and costly. Thus, data-driven methods are needed to accelerate the temperature estimation process while reducing the cost for the design and operation of the energy storage systems. However, most data-driven methods require a large training dataset to achieve the desired accuracy, resulting in enormous computational costs for finite element simulation. Therefore, an approach to building a high-fidelity surrogate model with less training data is needed. This research proposes a physics-informed machine learning framework to estimate the temperature of the pouch with the indirect liquid cooling system. The framework can achieve a high-fidelity temperature estimation with less training data by integrating the heat transfer partial differential equation into the loss function.

### 2.7. Dual-Scale Model Enabled Explainable-AI Toward Decoding Internal Short Circuit Risk of Lithium Metal Batteries

Jinrong Su * and Lei Chen
University of Michigan-Dearborn
* Correspondence: jinrongs@umich.edu

**Abstract:** The commercialization of lithium metal batteries (LMBs) is blocked by the dendrite-induced internal short-circuits (ISC). However, its risk assessment is hampered by trial-and-error testing and original structure-destructive-induced misleading data. Here, we develop an explainable physical-based data-driven framework, where the transparent assessment of Li dendrite-induced ISC risk is achieved from two aspects. In physics, a dual-scale model integrating microscopic lithium (Li) dendrite simulations with a macroscopic ISC model, thus enabling the interpretable connection among the internal microstructure evolution, the cell voltage, and ISC risk, which is not attainable by conventional cell-level ISC models without modeling internal states. In the artificial intelligence (AI) perspective, different from traditional machine learning (ML) models as a “black box”, explainable-AI (XAI) analyses over an ML-based ISC surrogate model can quantify both global and local insights into the importance of various factors in ISC risk. SHAP (SHapley Additive exPlanations) analysis identifies grain boundary defects and electrolyte thickness as the most influential factors, followed by charging rate, stack pressure, grain size, contact loss, and ionic conductivity. PDP (Partial Dependence Plots) provides local insights, revealing safety thresholds where higher grain boundary defects (>16.93 GPa), longer electrolyte thickness (>200 µm), charging rate near 0.91C, and grain size around 100 µm significantly mitigate ISC risks. The explainable, physical-based data-driven framework is general and readily customized to various batteries and energy systems.

### 2.8. Investigating the Effect of Thermophysical Inhomogeneity and Uneven External Cooling on Battery Degradation

Wenxue Liu *, Zhiwen Wan, Jason B. Siegel and Anna G. Stefanopoulou
University of Michigan
* Correspondence: wenxuel@umich.edu

**Abstract:** Understanding uneven electrothermal distribution inside the cell and its influence on battery degradation behavior from the first-principle perspective is of great importance for battery thermal designs and performance management. To this end, our work first proposes an electrochemical-thermal-degradation model to accurately simulate the spatiotemporal evolutions of battery internal states and parameters under typical testing profiles like constant-current discharging and urban drive cycles. This coupled model includes a single particle model with electrolyte, a distributed thermal model, and a mixed degradation model with three typical modes, which are coupled and solved on an open-source battery simulation platform, PyBaMM [1,2]. Second, the interplay between battery electrical, thermal, and aging behavior is thoroughly investigated by considering the parameter dependency of various submodels. The uneven electrothermal distribution due to inhomogeneous heat generation and heat transfer inside the battery is observed. The phenomenon is intrinsically attributed to the uneven current density distribution, the variations in thermophysical properties of battery materials, and inconsistent external cooling [3,4]. Finally, the essential influencing factors, including thermal conductivity, specific heat capacity, and convection coefficient, are examined. Their effects on battery inhomogeneous degradation are also comprehensively analyzed by considering parameter uncertainty, evidenced by the long timescale variations in local capacity and resistance, as well as in degradation-relevant electrochemical parameters. This work highlights the lasting effect of electrothermal maldistribution on battery inhomogeneous degradation and can provide significant insights into battery cell designs, thermal management system optimization, and health evaluation.

**References**

1. Sulzer, V.; Marquis, S.G.; Timms, R.; Robinson, M.; Chapman, S.J. Python Battery Mathematical Modelling (PyBaMM). *ECSarXiv* **2020**. https://doi.org/10.1149/osf.io/67ckj.
2. Pannala, S.; Movahedi, H.; Garrick, T.R.; Stefanopoulou, A.G.; Siegel, J.B. Consistently tuned battery lifetime predictive model of capacity loss, resistance increase, and irreversible thickness growth. *J. Electrochem. Soc.* **2024**, *171*, 010532.
3. Samad, N.A.; Wang, B.; Siegel, J.B.; Stefanopoulou, A.G. Parameterization of battery electrothermal models coupled with finite element flow models for cooling. *J. Dyn. Syst. Meas. Control.* **2017**, *139*, 071003.
4. Liu, W.; Hu, X.; Lin, X.; Yang, X.-G.; Song, Z.; Foley, A.M.; Couture, J. Toward high-accuracy and high-efficiency battery electrothermal modeling: A general approach to tackling modeling errors. *eTransportation* **2022**, *14*, 100195.

### 2.9. A Compact Hybrid Battery Thermal Management System for Enhanced Cooling

Jinrong Su * and Lei Chen
University of Michigan-Dearborn
* Correspondence: jinrongs@umich.edu

**Abstract:** Hybrid battery thermal management systems (HBTMS) combining active liquid cooling and passive phase change materials (PCM) cooling have shown a potential for the thermal management of lithium-ion batteries. However, the fill volume of coolant and PCM in hybrid cooling systems is limited by the size and weight of the HBTMS at high charge/discharge rates. These limitations result in reduced convective heat transfer from the coolant during discharge. The liquefaction rate of PCM is accelerated, and the passive cooling effect is reduced. In this paper, we propose a compact hybrid cooling system with multi-inlet U-shaped microchannels for which the gap between channels is embedded by PCM/aluminum foam for compactness. Nanofluid cooling (NC) technology with better thermal conductivity is used. A pulsed flow function is further developed for enhanced cooling (EC) with reduced power consumption. An experimentally validated thermal-fluid dynamics model is developed to optimize operating conditions, including coolant type, cooling direction, channel height, inlet flow rate, and cooling scheme. The results show that the hybrid cooling solution of NC + PCM + EC adopted by HBTMS further reduces the maximum temperature of the Li-ion battery by 3.44 °C under a discharge rate of 3C at a room temperature of 25 °Cwith only a 5% increase in power consumption, compared to the conventional liquid cooling method for electric vehicles (EV). The average number of battery charges has increased by about 6 to 15 percent. The results of this study can help improve the range as well as the driving safety of new energy EV.

### 2.10. Phase Change Material Seeded Coolant for Electronics Thermal Management

Yimin Zhou *, Tomasz Kulakowski and Solomon Adera
University of Michigan
* Correspondence: yiminzh@umich.edu

**Abstract:** Battery packs in electric vehicles (EVs), high-performance computing devices, and power conversion systems require efficient cooling to dissipate high heat fluxes within the limited volume and with minimal energy overhead. Conventional strategies like single-phase liquid cooling require high pumping power to maintain sufficient flow rates, while two-phase cooling methods, such as flow boiling, can suffer from flow instabilities, localized dry-out, and reliability issues caused by uncontrolled vapor formation and phase boundary migration. This work presents an innovative thermal management approach that integrates encapsulated phase change materials (PCMs) into a single-phase liquid coolant. The microencapsulated PCMs absorb excess thermal energy through melting, but unlike traditional two-phase systems, the phase change is confined within the capsules. This containment prevents the density-driven instabilities and abrupt vaporization events typically associated with boiling, ensuring smooth and predictable fluid behavior. To accommodate variable thermal loads, we further develop composite PCMs with tunable melting temperature ranges, enabling adaptive thermal buffering across diverse operating conditions. This modular and scalable design delivers enhanced heat removal while reducing pressure fluctuations, flow oscillations, and energy consumption. The proposed novel thermal management solution provides a compact, stable, and energy-efficient cooling strategy for next-generation batteries and high-power electronics, offering a robust alternative to conventional two-phase cooling methods.

### 2.11. Phase Change-Based Thermal Management for Battery Packs

Takiyah Ali * and Solomon Adera
University of Michigan
* Correspondence: takiyaha@umich.edu

**Abstract:** As of 2021, the transportation sector accounted for 28% of greenhouse gas emissions in the United States, the largest share of any economic sector. With current state-of-the-art battery-powered electric vehicles (EV) being more than six times energy efficient than conventional internal combustion engines, the environmental impact of vehicle electrification in our roadways is well-perceived. Despite the ecological benefits, electric vehicles come with a unique set of technological challenges, which pose barriers to their adoption by consumers. One such challenge is the thermal management of the battery cells and modules in electric vehicles. Lithium-ion (Li-ion) batteries, the most common battery type in electric vehicles, have an ideal operating temperature range of 15 to 35 °C. Operation outside the optimal temperature range not only reduces the available energy for driving the vehicle but also accelerates battery degradation. This necessitates efficient thermal management of battery packs, which can be achieved using phase change-based approaches. Due to the large latent heat, phase change cooling offers an extraordinary ability to suppress cell-to-cell, cell-to-pack, and pack-to-pack temperature variation. Here, we use metal additive manufacturing to create surfaces that enhance phase change boiling. With our additively manufactured novel surfaces that feature slanted pillars, we aim to increase not only the critical heat flux for boiling but also the heat transfer coefficient. The insights gained from this surface design can be integrated on the outer surfaces of battery packs for enhanced thermal management.

## 3. Session: Thermophysical Properties

### 3.1. The Hidden Flow: Unnoticed Rheology, Thermal Effects, and Their Impact on Daily Life, Industry, and Health

Leela Rakesh *
Central Michigan University
* Correspondence: lrakesh@aol.com

**Abstract:** Rheology quietly governs how we interact with the world—from squeezing toothpaste and pouring ketchup to walking on sand and stretching fabrics—through material behaviors like viscosity, elasticity, and flow transitions. Temperature subtly alters these properties, affecting performance, stability, and usability across a wide range of applications. This talk explores the often-overlooked thermal effects on rheological behavior in consumer products, industrial processes, pharmaceutical formulations, and biological fluids. We highlight how thermal rheology informs product design, drug delivery, and even disease progression through bedside rheometry. With advances in data capture and pattern recognition, it also paves the way for AI-driven tools in smart diagnostics, adaptive control, and material innovation. By connecting everyday experiences with advanced technologies, we show how thermal rheology bridges science and society across academic, healthcare, and industrial landscapes.

### 3.2. Building on History: 200+ Years of Thermal Analysis in Rubber Characterization

Gregory McKenna *
North Carolina State University
* Correspondence: gbmckenn@ncsu.edu

**Abstract:** Natural rubber, or *Hevea brasiliensis*, is a fascinating material that is one of our industrial society’s most important “enabling technologies”. Beyond the rubber tire that is essential to current automotive and commercial trucking applications, rubber is used in seals and gaskets, medical devices, personal protective equipment, and roofing materials all of which form the “underbelly” of our physical world. Here I will trace some of the history of the thermal analysis of rubbery materials which also formed an important basis for my own work, which I will also describe. Thermal analysis of rubber began with Gough’s [1] 1802 observations that “India” rubber or “Caoutchouc” gets warmer on stretching and cools upon release of the stretch. In 1859 Joule [2] made the observations quantitative using the newly invented thermo-multiplier, and he reported the first measurements of the heat capacity of India rubber, both uncross-linked and vulcanized. In the early 20th century Ruhemann and Simon [3] qualitatively commented on the relationship between stiffness and the “anomalous” calorimetric response of rubber at low temperatures and Bekkedahl [4] 1934 was the first to assign the idea of a second order transition to what we now call the glass transition event using dilatometric methods.

For my own work the Bekkedahl connection is profound as his dilatometer design was the inspiration for the classic work on structural recovery of glasses performed by Kovacs [5] and Bekkedahl also worked with the rubber group at the National Bureau of Standards where I held my first position in the 1970s. This abstract is not long enough to expand further on the specifics, but the talk will describe the early work and how it leads to my most recent works investigating very stable glasses as well as ongoing work related to developing alternative sources of natural rubber in the United States through an NSF funded Engineering Research Center named “TARDISS”.

**References**

1. Gough, J. A description of a property of caoutchouc, or Indian rubber. *Mem. Lit. Philos. Soc. Manch.* **1805**, *1*, 288–295. With some reflections on the elasticity of this substance Letter of November 16, 1802, read on February 11, 1803 published in Second Series.
2. Joule, J.P. On Some Thermo-Dynamic Properties of Solids. *Phil. Trans. R. Soc. Lond*. **1859**, *149*, 91–131.
3. Ruhemann, M.; Simon, F.Z. *Physikal. Chem. Abt. A* **1928**, *138*, 1–20.
4. Bekkedahl, N. J. *Res. Natl. Bur. Stand.* **1934**, *13*, 411–431.
5. Kovacs, A.J. *Fortschr. Hochpolym.-Forsch.* **1963**, *3S*, 394–507.

Acknowledgements: This material is based upon work supported by the U.S. National Science Foundation under Cooperative Agreement No. 2330145. Any opinions, findings and conclusions or recommendations expressed in this material are those of the author and do not necessarily reflect the views of the U.S. National Science Foundation.

### 3.3. Modeling the Glass-Liquid Transition in Thin Films: A Theoretical Approach Using the Modified Allen-Cahn Model

Koksal Karakus *, Leela Rakesh, Valeriy V. Ginzburg and Keith Promislow
Central Michigan University
* Correspondence: karakusk@gmail.com

**Abstract:** When conventional glasses are heated above their glass transition temperature, relaxation accelerates sharply, often unevenly due to thermal gradients and structural heterogeneity. In contrast, ultrastable glasses, prepared via vapor deposition, transform through a different mechanism: a mobility front emerges at the free surface and propagates inward at constant speed, governed by thermal stability and annealing temperature. We present a continuum model derived from free energy minimization, adapting the Allen-Cahn equation to describe the relaxation in both type of glasses. The potential function is calibrated to capture the mobility contrast and thermodynamic driving force, linking molecular-scale relaxation to mesoscopic front dynamics. While experiments support this behavior, a fully predictive continuum theory remains an open challenge. Key questions include how deposition temperature and speed affect front speed, how different glass formers behave, and whether a unified PDE framework can describe liquefaction across diverse materials.

### 3.4. Synchrotron X-Ray Mapping on Flow-Induced Crystallized Polymers

Xiaoshi Zhang *, Benson Jacob, Jason Alexander, Ryan Flanigan, Kirt Page, Arthur Woll, Ralph Colby and Alicyn Rhoades
Penn State—Behrend
* Correspondence: xvz5402@psu.edu

**Abstract:** Polymer crystallization under shear is a critical area of polymer research due to its significant impact on industrial processing techniques including injection molding and 3D printing. Under shear conditions, polymers become anisotropic and can develop complex structures such as shish-kebabs. Traditionally, the effect of shear on polymer structure has been studied using pressure-driven flow, which inherently includes pressure drop and shear gradient effects. In our research, we utilize a Couette flow setup in a rotational rheometer, allowing precise control over shear gradient, and sample size suitable for X-ray mapping. By employing synchrotron X-ray techniques at APS and CHESS, we capture a comprehensive view of the structural evolution under these conditions. This presentation will discuss our use of X-ray mapping to examine various responses in resin alignment in PEEK and its composites, the impact of melting temperature on the flow-induced crystallization of iPP, and how X-ray mapping enhances our understanding of iPP orientation as predicted by mold flow simulations.

### 3.5. Thermal Transport Characterization of Materials in Non-Ambient Conditions and Non-Conventional Configurations

Emma Mattinson * and Hitoshi Taniguchi
C-Therm Technologies Ltd.
* Correspondence: emma.mattinson@ctherm.com

**Abstract:** Thermal transport properties of a material change with the system temperature and pressure. Characterization of such properties is crucial to understanding the behavior of materials that undergo significant thermophysical transformation under different conditions, such as metal hydrides under hydrogen pressure. Furthermore, many characterization methodologies are limited by the sample requirements and configurations at which the measurement equipment must be installed to conduct measurements. For instance, investigation of localized thermal transport properties of a bulk material could be challenging with conventional testing setups.

The Trident Thermal Conductivity Analyzer offered by C-Therm Technologies offers different sensor modules that comply with non-ambient experimental conditions and configurations. This presentation will entail different applications in which Trident sensors have been employed for characterizations in non-ambient and non-conventional conditions, along with techniques employed to overcome traditional measurement limitations. The results demonstrate the versatility and robustness of the Trident platform in delivering accurate thermal transport data under challenging experimental setups, offering new insights for materials research and industrial applications.

## 4. Session: Advanced Instrumentation & Tandem Techniques

### 4.1. Undercooling Minimization in Ultrasound Coupled DTA Measurements of Molten Salts

Jacob Yingling *, Andrew Johanssen, Michael Woods and William Phillips
Idaho National Laboratory
* Correspondence: jacob.yingling@inl.gov

**Abstract:** Molten salts are ionic liquids that are used for the electrolytic pyroprocessing of metals and as heat transfer fluids in very high temperature processes. Recently, halide-based molten salt reactors (MSR) have gained momentum for high energy density and environmentally responsible electricity generation. The function of these reactors and their fuel cycle depend on a knowledge of the halide salt’s thermodynamic properties [1]. Therefore, high accuracy phase equilibria of MSR relevant base halide and actinide containing salts are needed.

Modern thermal analysis of the phase transitions of halide salts is usually done during heating at relatively high scan rates with commercial devices. Normally such measurements are adequate for pure or pseudo-binary salts. However, as the number of components in the mixture increases, accurately resolving the liquidus becomes increasingly difficult. Reversing the scanning mode greatly increases the sensitivity of phase transition measurements but can decrease accuracy due to undercooling [2]—a common occurrence in molten halide systems [3].

This work presents the development of a differential thermal analysis (DTA) cell intended for use in radiological gloveboxes. Non-contact ultrasonic agitation of the halide salt is implemented to enhance the kinetics of crystallization during cooling to minimize undercooling [4]. Measurements on halide salts are also presented to elucidate the effect of non-contact mixing on undercooling.

**References**

1. Boyd, S.; Taylor, C. 3—Chemical fundamentals and applications of molten salts. In *Molten Salt Reactors and Thorium Energy*; Dolan, T.J., Ed.; Woodhead Publishing: Stockton, UK, 2017; pp. 29–91. https://doi.org/10.1016/B978-0-08-101126-3.00003-8.
2. Nitsch, K.; Cihlář, A.; Rodová, M. Molten state and supercooling of lead halides. *J. Cryst. Growth* **2004**, *264*, 492–498. https://doi.org/10.1016/j.jcrysgro.2004.01.011.
3. Rycerz, L. Practical remarks concerning phase diagrams determination on the basis of differential scanning calorimetry measurements. *J. Therm. Anal. Calorim.* **2013**, *113*, 231–238. https://doi.org/10.1007/s10973-013-3097-0.
4. Zahir, M.H.; Mohamed, S.A.; Saidur, R.; Al-Sulaiman, F.A. Supercooling of phase-change materials and the techniques used to mitigate the phenomenon. *Appl. Energy* **2019**, *240*, 793–817. https://doi.org/10.1016/j.apenergy.2019.02.045.

### 4.2. Predicting Friction Material Composition Solely from TGA Data

Tina Adams *
The Lubrizol Corporation
* Correspondence: tina.adams@lubrizol.com

**Abstract:** A set of experimental friction materials with known, varied composition was used to develop analytical methods to quantify the amounts of individual components. The components in the friction material included fibers, particles, and resins and were typical in proportion of what might be used in a transmission.

The thermogravimetric (TGA) method was specifically designed to use kinetics of degradation of friction materials to our advantage and to obtain mass loss values that correlate to certain components. Thermal and thermo-oxidative traits of friction materials present a unique opportunity for modern and very precisely computer-controlled thermal analysis equipment.

Building more carefully on the observations monitored in this matrix, regression analysis was used with the thermogravimetry data from the full set of reference materials to transform mass loss for individual thermal events into the percentage of each material in the mixture. The resulting statistical models can be used to predict componentry in available test samples.

### 4.3. Dynamic Mechanical Analyzer Updated and Engineered for Comprehensive Viscoelastic Characterization of Materials

Peter Vichos *
NETZSCH Instruments North America, LLC
* Correspondence: peter.vichos@netzsch.com

**Abstract:** The NETZSCH DMA 303 Eplexor^®^ is the newest dynamic mechanical analyzer engineered for comprehensive viscoelastic characterization of materials across an extensive range of conditions. Offering a broad temperature spectrum from −170 °C to 800 °C and a controlled force range up to 50 N (both static and dynamic), it facilitates precise analysis of materials from soft polymers to rigid composites. The system’s high sensitivity, with a position resolution of 1 nm and frequency capabilities spanning 0.001 Hz to 150 Hz, ensures accurate detection of mechanical property variations. Real-time observation using a USB camera, protected by a fused silica light guide, permits live monitoring of sample deformation during testing Ergonomic and user-friendly design features such as height-adjustable sample holder areas, illuminated workspaces, and tool-free exchange of auto-detected sample holders using RFID enhance user comfort and overall operational efficiency.

### 4.4. Cooling Calibration of DSCs: Comparison of the Liquid Crystalline and Magnetic Transition Temperatures

Joseph Menczel *
Thermal Measurements LLC
* Correspondence: jmenczel@yahoo.com

**Abstract:** The temperature calibration of DSCs is usually done on heating, since only the melting point has reproducible values among transitions of traditional substances. Cooling calibration exists since the 90’s using liquid crystalline substances. Liquid crystals are organic substances, so their disadvantage is the low thermal stability when heated into the isotropic phase. Thermally stable liquid crystals of Merck (CE-3 and CE-8) that were ideal for the cooling calibration, are not available anymore, and the liquid crystals proposed in ASTM 2069 (M-24, HP-53 and BCH-52) exhibit a ca. 0.4 °C decrease in the nematic-to-isotropic transition temperature after just one heating. This transition temperature decrease is due to slight thermal degradation, therefore new substances had to be found for carrying out cooling calibration with an accuracy of ~±0.1 °C. Such transitions were found in the Curie and Néel transitions ferromagnetic and ferrimagnetic substances. Alumel and nickel seem to be optimum materials for the cooling calibration. Comparison of the correction factor (see Menczel and Leslie, 1990, 1993) determined from the nematic-to-isotropic temperature of liquid crystals CE-3 and CE-8 indicated that the transition of the ferromagnetic-to-paramagnetic transition of ferromagnetic materials has no supercooling, and can be used for temperature calibration of DSCs in both heating and cooling modes to an accuracy better than ±0.1 °C. In addition, the use of ferromagnetic materials can make up for the absence of calibrating standards even in the heating mode in the low and room temperature regions, like EuO (−204 °C), Dy (185 °C), FeCl2 (24 °C), CrCl2 (25 °C) and NiCl2 (50 °C).

**References**

1. Menczel, J.D.; Leslie, T.M. Temperature calibration of a power compensation DSC on cooling. *Thermochim Acta* **1990**, *166*, 309–317.
2. Menczel, J.D.; Leslie, T.M. Temperature calibration of an electrical compensation DSC on cooling using thermally stable high purity liquid crystals. *J. Therm. Anal.* **1993**, *40*, 957–970.

### 4.5. Solid Water Interactions and Their Impact on Storage, Processing, and Shelf Life of Materials

Matthias Wagner *, Markus Schubnell, Nicolas Fedelich, Andreas Bach and Angela Hammer
METTLER TOLEDO
* Correspondence: matthias.wagner@mt.com

**Abstract:** Interactions between solid materials and water have profound effects on the storage, processing, and shelf life of materials across diverse industries. These solid–water interactions, including adsorption, absorption, and phase changes, can alter the physical and chemical properties of materials, impacting their stability, integrity, and functionality. Understanding these interactions is crucial for optimizing storage conditions to prevent degradation, refining processing techniques to maintain material flow, and enhancing shelf life by mitigating moisture-induced deterioration. This work discusses current research methods and explores practical applications in sectors such as food technology, pharmaceuticals, and materials science.

### 4.6. Introducing the TGA Smart-Seal Pans™

Carlton Slough *
TA Instruments
* Correspondence: gray_slough@waters.com

**Abstract:** Many advanced materials, for example battery and pharmaceutical, are highly reactive with atmospheric components, including oxygen and water vapor. Not only handling but testing of such materials must be done in an inert environment, such as a glovebox. Installing test instrumentation in gloveboxes, however, is counterproductive on many levels. Therefore, testing methods that do not require a glovebox have advantages over those that do. Thermogravimetric analysis (TGA) is an essential tool in the study of materials, providing information on stability, composition, and degradation characteristics. However, the technique presents unique difficulties, as the sample must be in an open container when analyzed so that gases can evolve freely. Standard TGA sealed pans offered by manufacturers often fail to protect the most sensitive samples as they are opened and exposed to the ambient atmosphere prior to loading. This talk introduces a new type of hermetic TGA sample pan: the TGA Smart-Seal Pan™. This pan, using shape-memory technology, automatically opens inside the inert TGA furnace environment, thereby providing testing capabilities without the need of a glovebox.

## 5. Session: Composites and Nanocomposites

### 5.1. Mechanical Reinforcement in Polymer Nanocomposites: Insights from Small-Angle Neutron Scattering

Yangyang Wang * and Shiwang Cheng
Oak Ridge National Laboratory
* Correspondence: wangy@ornl.gov

**Abstract:** Hydrodynamic effects play a fundamental role in the mechanical reinforcement of polymer nanocomposites (PNCs). However, the influence of nanoparticles on the deformation and relaxation mechanisms of polymers has not been fully understood. This talk reviews recent progress [1,2] on understanding the microscopic origin of mechanical reinforcement in polymer nanocomposites by using small-angle neutron scattering (SANS). By taking advantage of the zero-average-contrast method, the single-chain structure factor of the polymer in deformed polymer nanocomposites can be directly determined by SANS. Rheological experiments on silica filled poly(methyl methacrylate) and polystyrene samples show that the mechanical properties in these systems are enhanced by the presence of nanoparticles through the hydrodynamic reinforcement mechanism. On the other hand, SANS measurements reveal no enhanced structural anisotropy in the PNCs, compared with the pristine polymers under identical deformation conditions. These results suggest that the mechanical reinforcement in these PNCs is not a result of molecular overstraining but originates from a redistribution of the strain field in the polymer matrix. Additionally, under large macroscopic deformation and local disturbance field, the nonlinear response of entangled polymers and the nonlinear relation between polymer structural anisotropy and microscopic strain lead to accelerated relaxation dynamics and reduced structural anisotropy. These findings help to clarify the molecular origin of the mechanical reinforcement in deformed PNCs and provide a new perspective for understanding the influence of nanoparticles on the structure and dynamics of their surrounding polymer phase.

**References**

1. Sun, R.; Melton, M.; Safaie, N.; Ferrier, R.C., Jr.; Cheng, S.; Liu, Y.; Zuo, X.; Wang, Y. Molecular view on mechanical reinforcement in polymer nanocomposites. *Phys. Rev. Lett.* **2021**, *126*, 11780. https://doi.org/https://doi.org/10.1103/PhysRevLett.126.117801
2. Sun, R.; Yang, J.; Patil, S.; Liu, Y.; Zuo, X.; Lee, A.; Yang, W.; Wang, Y.; Cheng, S. Relaxation dynamics of deformed polymer nanocomposites as revealed by small-angle scattering and rheology. *Soft Matter*. **2022**, *18*, 8867. https://doi.org/https://doi.org/10.1039/D2SM00775D

### 5.2. Dynamics and Rheology of Polymer Nanocomposites with Repulsive Polymer-Nanoparticle Interactions

Neda Hashemipour * and Shiwang Cheng
Michigan State University
* Correspondence: hashemip@msu.edu

**Abstract:** Polymer nanocomposites are promising lightweight materials with broad applications in energy, environmental, and healthcare technologies. While many studies have focused on systems with attractive polymer–nanoparticle interactions, the role of repulsive interactions remains largely unexplored. In this work, we synthesized surface-functionalized titanium dioxide (TiO_2_) nanorods designed to exhibit strong repulsive interactions with poly(vinyl acetate) (PVAc), serving as the polymer matrix. Small-angle X-ray scattering (SAXS) reveals pronounced aggregation of TiO_2_ nanorods and the formation of a nanoparticle network, consistent with the presence of strong repulsive interfacial interactions. Surprisingly, broadband dielectric spectroscopy (BDS) and differential scanning calorimetry (DSC) show an accelerated structural relaxation and a reduced glass transition temperature (Tg) in the nanocomposites. Despite these dynamic changes, rheological measurements indicate minimal changes in the linear viscoelastic behavior compared to neat PVAc. These findings demonstrate that repulsive polymer–nanoparticle interactions can significantly influence dispersion, segmental dynamics, and glass transition without substantially affecting linear rheology, offering new insights into interfacial design strategies for polymer nanocomposites.

### 5.3. The Effect of Nanotube Length and Functionalization on Rheological Properties of Carbon Nanotube/Polymer Composites

Brian Grady * and Mason Rhue
University of Oklahoma
* Correspondence: bpgrady@ou.edu

**Abstract:** In our lab we have synthesized ultralong multiwalled carbon nanotubes with purity and diameter similar to commercial high-quality tubes using exfoliated planar vermiculite as the growth surface which enables the production of lower cost nanotubes since the yield (pounds of nanotubes/pounds of catalyst + support) is very high. However, dispersing such tubes into polymers is a significant challenge due to very high melt viscosity as well as the vertical alignment of tubes that results from the synthetic process. The length of the tubes can be reduced using ball milling. As will be showed, milling alone improves the ability to disperse as measured by oscillatory rheology, but the dispersion is still substandard compared to the dispersion of typical commercial tubes. Hence, various chemical treatments during ball milling were applied and results were compared. Two different polymers were used, polycarbonate and a rubber corresponding to a tire tread formulation. Overall, the results show some promise but even with functionalization, these ultralong tubes did not disperse very well.

### 5.4. Dynamics of Poly(Methyl Acrylate)/Poly(Methyl Methacrylate)-Grafted Fe_3_O_4_

Shalin Patil *, Christopher Mbonu, Tsengming Chou, Ruhao Li, Di Wu, Pinar Akcora and Shiwang Cheng
Michigan State University
* Correspondence: patilsh5@msu.edu

**Abstract:** We investigated the dynamics of nanocomposites prepared through mixing poly(methyl methacrylate) grafted Fe_3_O_4_ nanoparticles (PMMA-g-Fe_3_O_4_) in poly(methyl acrylate) (PMA). A key feature here different from previous dynamics measurements of polymer nanocomposites is the different chemistry between the matrix polymer and the polymer grafts, which introduces chemical heterogeneity. Transmission electron microscopy shows clear evidence of nanoparticle clustering due to the poor miscibility between the bulk PMA and the bulk PMMA. At the same time, broadband dielectric spectroscopy measurements detect two leading relaxations, i.e., the α and α* processes, where the α process is associated with the bulk PMA and the α* process from the PMA interacting with the grafted PMMA in the nanoparticle clustering region. Interestingly, the characteristic relaxation time of α*, τα*, is slightly slower than that of the α, τα, at high temperatures, and exhibit nearly Arrhenius temperature dependence at low temperatures. As a result, the τα* and τα cross each other in the activation plot upon cooling and the τα* s observed at temperatures approaching the glass transition temperature of PMA. Our observations suggest the presence of component dynamics and dynamics confinement effect between PMA and PMMA in the nanoparticle clustering region, highlighting an active interaction between PMA and PMMA at the interface despite their poor miscibility. These results thus suggest new routes to control interface dynamics through immiscible polymer pairs.

### 5.5. Spatial Gradient of the Dynamics of Capped Thin Polymer Films Through Broadband Dielectric Spectroscopy

Juncheng Zheng * and Shiwang Cheng
Michigan State University
* Correspondence: zhengju7@msu.edu

**Abstract:** Polymers exhibit distinct dynamical behavior in the vicinity of interfaces compared to their bulk counterparts away from the interface. However, the nature of the spatial gradient of near-interface dynamics remains unresolved. The alternation of polymer dynamics is typically confined to the nanoscale region adjacent to interfaces, posing significant experimental challenges in their characterization. In this work, we quantify the interfacial dynamics gradient in capped polymer thin films using broadband dielectric spectroscopy. The spatial gradient of dynamics is quantitatively deconvoluted employing our new analysis, the continuous Havriliak-Negami (HN) function model. We present the evolution of the near-interface dynamics gradients across a wide range of capped film thicknesses, from 100 nm down to below 10 nm. The influence of nanoconfinement on the spatial dynamics gradient is investigated, and its comparison with existing theoretical models and experimental observations will also be discussed.

### 5.6. Recycling Carbon Fiber Epoxy Composites via Lewis Acid-Mediated Solvolysis

Cassandra Reese *, Wenbin Kuang and Kevin Simmons
PNNL/Bat
* Correspondence: cassandra.reese@pnnl.gov

**Abstract:** Carbon fiber-reinforced polymer (CFRP) composites have gained widespread adoption across industries ranging from aerospace and automotive to energy and sporting goods due to their high strength-to-weight ratio and durability. However, the thermoset matrices that enable CFRPs’ exceptional mechanical performance also hinder their end-of-life recycling. Their highly crosslinked structures resist degradation, limiting recyclability and contributing to growing volumes of composite waste. Meanwhile, the production of virgin carbon fiber is both energy- and emissions-intensive, driving the need for recycling strategies that can efficiently recover and reuse both high-value fibers and matrix materials within manufacturing workflows.

This work presents a chemical recycling process that enables the recovery of both carbon fibers and matrix polymer fragments from amine-cured epoxy CFRPs using a Lewis acid-buffered solvolysis system. The approach promotes selective C–N bond cleavage under moderate temperatures and can be performed under both autoclave and ambient pressure conditions. Recovered carbon fibers retain over 90% of their original mechanical properties, while recycled oligomers offer potential for reuse.

Current efforts focus on optimizing the recycling process, improving the recovery and washing of recycled carbon fiber, and developing strategies for the recovery and purification of both the catalyst and recycled oligomers. This approach offers a viable path toward circular CFRP material flows and second-life applications across transportation, energy storage, and infrastructure.

### 5.7. Carbon Precursor Resins and Semi-Fluorinated Polyaryl Ethers. Synthesis, Performance, and Commercialization

Ernesto Borrego ^1,2^, Gustavo Munoz ^1,2^, Will Johnson ^1^, Niroshani Abeynayake ^1,2^, Maxim Solovyev ^1^, Erfan Masaeli ^1^, Charles Pittman, Jr. ^1^ and Dennis W. Smith, Jr. ^1,2,^*
^1^ Mississippi State University
^2^ Hand Technologies, LLC
* Correspondence: dsmith@chemistry.msstate.edu

**Abstract:** The demand for carbon fiber matrix composites wherein the matrix materials are chosen from carbon and/or ceramic elements formed by CVD or pyrolysis of suitable precursors has led the application development of carbon precursor resins. Although commercial methods based on carbonization of phenolic resins have produced carbon-carbon composites (C/C) for 50+ years, production rates demanded now are severely limited due to low carbon yield, multiple re-infusion steps, and slow carbonization requirements. In addition, the delivery of variable geometry graphitic carbon at the micro- and nanoscale with controlled crystallinity is highly sought due to the energy and information revolutions currently underway. To address these demands, our collaborative laboratory is developing resin technology based on the highly efficient thermal (Bergman) cyclopolymerization and copolymerization of bis-o-diynylarene (BODA) monomers affording intermediate reactive resins which can be melt processed, thermally cured to polyarylene networks, and pyrolyzed (1000 °C) to high yield (83%) carbon-carbon composites [1]. In addition, specialty semi-fluorinated aromatic ether polymers from step-growth polymerization of (1) fluoroalkenes, and (2) aryl ethers and hexafluoroacetone hydrate (via EAS) is presented. Well established perfluorocyclobutyl (PFCB) aromatic ether polymers and related architectures have demonstrated application as low k insulators, PEM fuel cells, hosts for emissive or electro-optic chromophores, optically tunable films (low loss, variable RI) with high thermal stability and thermally crosslinkable without the use of post-curatives [2–8]. An overview of both material platforms and commercialization efforts is presented.

**References**

1. Borrego, E.; Athukorale, S.; Gorla, S.; Duckworth, A.; Johnson, W.; Kundu, S.; Toghiani, H.; Farajidizaji, B.; Pittman, C.; Smith, D.W., Jr. High Carbon Yielding and Melt Processable Bis-ortho-Diynylarene (BODA)-Derived Resins for Rapid Processing of Dense Carbon/Carbon Composites. *Compos. Part B* **2022**, *242*, 110080. And references therein. BODA Resin samples are available from Hand Technologies, LLC (handtechllc.com).
2. Park, J.; Kucukkal, T-G; Oh, J-M; Stuart, S.; Smith, D.W., Jr.; Creager, S.E. Selective Copolymerization and Post-Sulfonation toward Segmented Perfluorocyclobutyl (PFCB) Aromatic Ether Copolymers. *Polym. Chem.* **2024**. https://doi.org/10.1039/D4PY00149D.
3. Munoz, G.; Chamberlain, K.; Athukorale, S.; Pittman, C.U., Jr.; Smith, D.W., Jr. Semi-fluorinated (6F) Polyaryl Ethers and Polyphenylenes via Interfacial Electrophilic Condensation Polymerization with Hexafluoroacetone Hydrate. *Polymer* **2024**, *293*, 126642.
4. Iacono, S., Weeks, N.; Smith, D.W., Jr. Systematic Study of Nucleophile Additions to Aryl Trifluorovinylethers: A Versatile Route toward Fluorinated Ether Intermediates of Synthetic Interest. *J. Fluor. Chem.* **2023**, *271*, 110187.
5. Munoz, G.; Chamberlain, K.; Athukorale, S.; Ma, G.; Gu, X.; Pittman, C.U., Jr.; Smith, D.W., Jr. Teaching Old Polymers New Tricks. Improved Synthesis and Anomalous Crystallinity for a Lost Semi-Fluorinated Polyaryl Ether via Interfacial Polymerization of Hexafluoroacetone Hydrate and Diphenyl Ether. *Macromol. Rapid Commun*. **2023**, *44*, 200737.
6. Faradizaji, B.; Borrego, E.I.; Jazi, M.E.; Smith, D.W., Jr. Triphenylene-Enchained Perfluorocyclobutyl (PFCB) Aryl Ether Polymers. A Modular Synthetic Route to Processable Thermoplastics Approaching Upper Limit Tg and Photostability. *Macromolecules* **2021**, *54*, 7666.
7. Shelar, K.; Mukeba, K.; Mills, K., Smith, D.W., Jr. Renewable Isosorbide-Containing Semi-Fluorinated Aromatic Ether Polymers. *J. Polym. Sci. Part. A. Polym. Chem*. **2022**, *60*, 2500–2507.
8. Mukeba, K.M.; Shelar, K.E.; Faradizaji, B.; Borrego, E.I.; Caldona, E.B.; Pittman, C.U., Jr.; Smith, D.W., Jr. Semi-fluorinated Poly(aryl ether sulfone)s via Step-Growth Polymerization of Perfluorocyclohexene with Bisphenols. *Polymer* **2022**, *253*, 124937.

### 5.8. Network Dynamics of Polymer Nanocomposites

Shiwang Cheng *
Michigan State University
* Correspondence: chengsh9@msu.edu

**Abstract:** The dynamics of polymer nanocomposites are dictated by two intertwining components: the polymer matrix and the nanoparticle network. Compared with the extensive effort in characterizing the structural relaxation and chain diffusion of the polymer matrix, the dynamics of the nanoparticle network remain largely unknown. In this contribution, we combine dielectric spectra and rheology to investigate the network dynamics and their influence on the viscoelastic properties of polymer nanocomposites. In particular, this presentation focuses on addressing the following questions: (i) What are the characteristics of dynamics from nanoparticle networks? (ii) How are the dynamics of the nanoparticle network coupled with the dynamics of the polymer matrix? (iii) How do the polymer-nanoparticle interactions affect the nanoparticle network dynamics? Although the contribution focuses primarily on experiments from linear viscoelastic measurement and recent dielectric measurements, the structural evolution of the nanoparticle network and its impact on the mechanical properties of polymer nanocomposites under large deformation will also be discussed.

## 6. Session: Kinetics

### 6.1. Stability and Crystallization of Indomethacin/Sucrose Benzoate Co-Amorphous Glasses

Elaheh A. T. Moghadam * and Sindee Simon
NC State University
* Correspondence: eabdoll@ncsu.edu

**Abstract:** Isothermal crystallization of indomethacin and binary miscible mixtures of indomethacin and sucrose benzoate samples are investigated from a super-cooled liquid state using differential scanning calorimetry to study the stability of the systems. These mixtures, classified as co-amorphous systems, are known to demonstrate advantages such as enhanced water solubility and bioavailability relative to the crystalline active pharmaceutical ingredients. The crystallization data for indomethacin as a function of time at different temperatures were modeled using the Avrami equation. The derived data are also used to obtain the time-temperature-transformation (TTT) diagram, which is fitted to the nucleation rate theory model. Results revealed that increasing sucrose benzoate content improved the system’s resistance to crystallization. The composition with 49% sucrose benzoate exhibited the highest stability, representing the most stable co-amorphous formulation in this study.

### 6.2. Dependence of Curing Kinetics on Mass Ratio for Epoxy/Amine Systems and on Intensity of UV Light for Photopolymers

Elena Moukhina * and Claire Strasser
NETZSCH Geraetebau GmbH
* Correspondence: elena.moukhina@netzsch.com

**Abstract:** Kinetics of curing reactions depend not only on temperature but on additional factors presented in these processes only.

The reaction in a two-component chemical system depends on both temperature and the mass ratio of reactants. Kinetic analysis of the reaction rate for given mass ratio and different temperature conditions provides a kinetic triplet with kinetic parameters for each reaction step. Changing the mass ratio leads to the changing of kinetic parameters in the kinetic triplet. The materials can contain some additives or solvents, and sometimes the molar ratio is unknown and cannot be calculated. Additionally, for some reactions the molar masses and stoichiometry are also unknown. Therefore, the classical methods based on known molar masses, stoichiometry and concentrations cannot be applied for kinetic analysis in this case.

Firstly, the analytical dependences on mass ratio were found for apparent kinetic parameters of the kinetic triplet such as reaction order, pre-exponential factor, pre-exponent of autocatalysis, and reaction enthalpy. These dependences are used in the new method for common kinetic analysis of the experimental data measured at different temperature conditions and different initial mass ratio of reactants. The method allows for fast creating the common kinetic model depending on both temperature and mass ratio.

The second analyzed factor is the intensity of UV light for reactions in photopolymers. The reaction rate increases with the light intensity. The common kinetic model is created where curing rate depends on both temperature and intensity of UV light. The model is applied for acrylate photopolymers in additive manufacturing.

Both methods are implemented in NETZSCH Kinetics Neo software and verified for the analysis of the multi-step cross-linking process with different mass ratios of epoxy/amine as well as for photopolymerization of acrylate photopolymers under different intensities of UV light.

### 6.3. Combined Effects of Temperature and Humidity on the Decomposition Kinetics of Nitrocellulose-Based Propellants: From Milligram to Kilogram Scale

Bertrand Roduit *, Patrick Folly, Alexandre Sarbach and Richard Baltensperger
AKTS SA
* Correspondence: b.roduit@akts.com

**Abstract:** This study presents the results of investigations into the combined impact of temperature and humidity on the chemical stability of nitrocellulose (NC)-based propellants, widely used in munitions. Three complementary experimental techniques were employed to characterize the decomposition kinetics:

Differential Scanning Calorimetry (DSC) under non-isothermal conditions (heating rates from 0.25 to 4 K/min) to assess overall decomposition at elevated temperatures (up to 260 °C) over short timescales.

Heat Flow Calorimetry (HFC) under isothermal conditions (60 to 100 °C), a highly sensitive method that enables precise monitoring of the early stages of degradation over extended durations at temperatures close to real-world storage conditions.

Monitoring of stabilizer consumption, a key chemical indicator of aging in NC-based formulations.

By combining these techniques, the study enables accurate and robust kinetic modeling of the decomposition process under realistic environmental conditions, whether defined by the STANAG 2895 standard or recorded via onboard environmental sensors (dataloggers). A key advancement of this work is the development of a kinetic model that integrates the effects of both temperature and absolute humidity on the reaction course. While temperature is a well-established driver of chemical aging, the inclusion of humidity significantly enhances the realism and predictive power of the kinetic analysis.

Presented extended kinetic approach highlights the strong accelerating effect of moisture on degradation, as evidenced by faster stabilizer depletion and increased heat release measured via HFC. Incorporating humidity provides a more comprehensive understanding of the aging process, particularly in dynamic and humid climates.

The derived kinetic parameters enable reliable predictions of critical safety metrics across a broad mass scale, including:

Self-Accelerating Decomposition Temperature (SADT).

Time to Maximum Rate under adiabatic conditions (TMRad).

Ignition temperature in slow cook-off tests, across a wide range of temperature and absolute humidity conditions (corresponding to relative humidity levels from 0% to 95%, normalized to 25 °C for consistency).

The excellent agreement between experimental data and model predictions confirms the robustness of this approach for forecasting the long-term behavior of NC-based propellants, from small laboratory samples to full-scale systems. Moreover, the kinetic analysis developed here, by incorporating humidity as an additional dynamic variable, can be adapted to explore the influence of other environmental or chemical factors beyond temperature alone, broadening its applicability in the field of energetic material aging and safety assessment.

### 6.4. Action of Organophosphorus Flame Retardants: Impact of Structure and Mode of Thermal Degradation

Bob A. Howell *
Central Michigan University
* Correspondence: bob.a.howell@cmich.edu

**Abstract:** Polymeric materials have been essential to the development of modern societies around the world. While these materials have brought major benefits, they are, in the main, quite flammable. Flammability must be controlled to permit most uses. This is most often done by incorporation of a flame retardant additive. While a wide range of materials, usually selected empirically, have been used for this purpose, organohalogen and organophosphorus compounds have been most useful. Traditionally, organohalogen compounds, particularly brominated aryl ethers, have been widely used. They are effective gas-phase flame retardants, are readily available, and inexpensive. However, they easily leach from matrices in which they have been incorporated, persist in the environment, tend to bioaccumulate, and pose human health risks. Consequently, they are being replaced by organophosphorus compounds, which are generally much less toxic. In particular, phosphorus compounds derived from renewable, readily available, inexpensive, nontoxic biobased precursors are being developed. These compounds, depending on structure, may be either solid-phase active (promoting the formation of an insulating char layer at the surface of a degrading polymer matrix) or gas-phase active (liberating reactive species to scavenge propagating radicals in the combustion zone). The origin of this difference lies in structural features that determine the mode of thermal decomposition in a degrading polymer matrix. Compounds containing phosphorus at a high oxidation level, e.g., phosphates, decompose at relatively low temperatures to form phosphorus acids, which promote char formation. Compounds containing phosphorus at low oxidation level, e.g., phosphonates, phosphinates, ultimately decompose in a degrading polymer matrix at higher temperature to generate PO radicals, some of which avoid reaction with matrix components and escape to the gas phase where they act as efficient scavengers of combustion propagating radicals.

### 6.5. Kinetic Modelling of Pressure Influences on Reactions in Solids

Elena Moukhina *
NETZSCH Geraetebau GmbH
* Correspondence: elena.moukhina@netzsch.com

**Abstract:** Reaction kinetics depends on the temperature conditions. The kinetic model, based on laboratory measurements, allows for the simulation of the reaction progress at user-defined temperature conditions. The simulation is possible for lifetime predictions, for the study of thermal stability for safe storage, as well as for the optimization of the temperature profile in industrial applications like polymers, food, pharmaceuticals, or ceramic industries to reduce costs and improve the quality of the final products.

However, many reactions depend not only on temperature, but on additional parameters like humidity, or the presence of the active component in the surrounding atmosphere and its partial pressure. For example, enhancing oxygen content increases the rate of oxidation reactions, and increasing the hydrogen concentration increases the rate of reduction reactions.

Another mechanism is present during reversible decomposition, where the presence of the active component in the surrounding atmosphere increases the reverse reaction only. The overall rate of reversible decomposition depends on the partial pressure of the product in the surrounding atmosphere, like decomposition with water release in the humid atmosphere, or decomposition with release of carbon dioxide in the atmosphere under different partial pressures of carbon dioxide.

Last considered mechanism is the influence of pressure of inert gas, which influenced indirectly on product concentration during reversible decomposition. The work presents examples of all these mechanisms and kinetic models created in NETZSCH Kinetics Neo software.

## 7. Session: Sustainable Polymers

### 7.1. Sustainable 3D-Printing Materials: Printability and Performance

Michael Toomey *
Oak Ridge National Lab
* Correspondence: toomeymd@ornl.gov

**Abstract:** We report the utilization of plant biomass fraction, lignin, as a feedstock for the preparation of new bio-based 3D-printing materials. 3D-printing of a material using fused deposition modeling, a layer-by-layer deposition-based structure building from the molten material through a printer nozzle, requires understanding of the material’s rheological and thermal properties. To improve the material throughput and printability, the hurdle of lignin melt-processing needs to be addressed. We discovered the chemistry of selected lignins and their molecular rigidity to prepare highly loaded lignin (50 wt%) composites with tunable flow characteristics. Time-temperature-superposition was applied to investigate the materials’ rheological properties within a wide range of temperature and angular frequency. A window of viscosity and shear rate was determined for good printability. Apart from lignin thermoplastic blends, new compositions based on lignin-based sustainable vitrimers are being developed for the manufacturing of repairable parts via high-shear processing and 3D printing. The incorporation of reinforcing fiber enhances the toughness of the printed or molded composites. The mechanical testing indicated high performance of the printed objects with the presence of the fillers.

### 7.2. Pressure-Differential Scanning Calorimetry of Virgin and Recycled Polyethylene

Heather Snell *
Intertape Polymer Group
* Correspondence: hsnell@itape.com

**Abstract:** Companies are moving towards using more sustainable materials for the manufacturing of products with the goal of becoming more environmentally friendly. One way to work towards this goal is to use recycled material versus virgin material to prevent the recycled material from going to a landfill after use. In this case, polyethylene is the material of interest. There are two types of recycled polyethylene, post-consumer recycled (PCR) and post-industrial recycled (PIR). PCR is waste that has been discarded by consumers into the waste stream and then recycled [1]. PIR recycled material is a by-product of a manufacturing process coming directly from the processing that is then used within the same facility [2]. When a company receives PCR and PIR materials, there is no information on how much antioxidant or other additives are present, as the material comes from different sources. The amount of antioxidant activity can be measured by High Pressure Differential Scanning Calorimetry (P-DSC). This is important because differences in the amount of antioxidants present could affect the processing of the resin and lead to manufacturing issues.

Samples of virgin, PCR, and PIR polyethylene resin pellets were analyzed by P-DSC. For the P-DSC analysis, the samples were exposed to a 150-psi oxygen atmosphere, heated at 10 °C/min to 190 °C, and held isothermally until a sudden change in the signal was detected in the thermogram [3].

No correlation could be seen between the virgin, PCR, and PIR samples; there was variation in all of the samples and groups. However, this information gives information on the comparative amount of antioxidants present in a resin pellet. This information can help the engineering team determine if the method of processing the polyethylene resin pellets needs to be adjusted for antioxidant content.

**References**

1. Klingenberg, P.; Schirmeister, C.; Kappeler, M.; Calean, A.; Biester, H.; Licht, E.; Rapp, B. Quantification of Regulated Metals in Recycled Post-Consumer Polypropylene through Comparative ICP-MS, AAS and Libs Analyses. *Polym. Test.* **2024**, *136*, 108480. https://doi.org/10.2139/ssrn.4767360.
2. Arese, M.; Bolliri, I.; Ciaccio, G.; Brunella, V. Post-industrial recycled polypropylene for automotive application: Mechanical properties after thermal ageing. *Processes* **2005**, *13*, 315. https://doi.org/10.3390/pr13020315.
3. Šimon, P.; Fratričová; M; Schwarzer, P.; Wilde, H.-W. Evaluation of the residual stability of polyurethane automotive coatings by DSC. *J. Therm. Anal. Calorim.* **2006**, *84*, 679.

### 7.3. Go with the Flow: Viscoelastic Behavior of Biopolymer-Based Rheology Modifiers in Today’s Complex Fluids

Jin Zhao *, Matthias Knarr, and Leela Rakesh
Roquette
* Correspondence: jin.zhao@roquette.com

**Abstract:** Applied rheology plays a pivotal role in the formulation and performance optimization of complex fluids and soft materials, particularly in industries such as pharmaceuticals, food, cosmetics, and construction. Among the most widely used rheology modifiers are natural and semi-synthetic polysaccharides, including cellulose ethers (e.g., hydroxypropyl methylcellulose, carboxymethyl cellulose) and alginates. These biopolymers exhibit unique viscoelastic properties that are highly sensitive to concentration, molecular weight, ionic strength, and temperature, enabling precise control over flow behavior and texture. This talk reviews the rheological behavior of aqueous systems containing cellulose ethers and alginates, focusing on their shear-thinning characteristics, gelation mechanisms, and synergistic interactions with other hydrocolloids and salts. Through steady, dynamic shear and other industrially relevant testing, we demonstrate how formulation parameters influence viscosity, yield stress, and thixotropy, providing insights into their application-specific performance. The extended research findings in this area underscore the importance of tailoring rheological profiles to meet functional requirements in diverse applications, from enhancing spreadability in topical formulations to improving pumpability and sag resistance in construction materials.

### 7.4. Unraveling the Influence of Hydrogen and Blended Gas on Polymer Performance in Infrastructure Systems

Wenbin Kuang *, Kevin Simmons, Nalini Menon and Logan Kearney
Pacific Northwest National Laboratory
* Correspondence: wenbin.kuang@pnnl.gov

**Abstract:** Polymeric materials are vital for sealing and barrier functions in hydrogen and natural gas systems, yet their long-term compatibility with hydrogen is poorly understood. The H-Mat and HyBlend projects, funded by the Department of Energy, aim to address this knowledge gap. We explore how hydrogen and hydrogen-methane blends affect polymeric and elastomeric materials, using diverse characterization and testing methods. Research spans pressures from 100 to 14,000 psi, focusing on distribution pipeline materials and rubber systems. Our findings aim to guide the design and maintenance of infrastructure for hydrogen and blended gas technologies, enhancing safety and reliability while advancing sustainable energy solutions globally.

### 7.5. Advancing Sustainability: Thermal Analysis of PLA Composites

**Karim Elhattab** *
TA Instruments
* Correspondence: karim_elhattab@waters.com

**Abstract:** The rapid evolution of the sustainable materials industry requires thorough characterizations to ensure optimal properties for diverse applications. Thermal analysis techniques are pivotal in this advancement, offering critical insights into material structure, behavior, and processing. This presentation will delve into the significance of thermal analysis in advancing sustainable materials, focusing on the well-known polylactic acid (PLA) and its composites.

### 7.6. Thermal Characterization of Devulcanized Ground Tire Rubber (dGTR): Advancing Sustainability and the Circular Economy

Cathy Stewart *
Intertape Polymer Group
* Correspondence: cstewart@itape.com

**Abstract:** The accumulation of end-of-life tires presents an ongoing environmental challenge, driving the need for efficient recycling strategies. Devulcanized ground tire rubber (dGTR) has emerged as a promising alternative to virgin rubber, offering opportunities for reuse in various industrial applications. This study employs thermal analysis techniques—including thermogravimetric analysis (TGA), differential scanning calorimetry (DSC), and dynamic mechanical analysis (DMA)—to evaluate how the devulcanization process influences material properties. TGA assesses thermal stability and compositional changes, DSC examines alterations in glass transition temperature and potential curing behavior, while DMA provides insight into viscoelastic characteristics like storage modulus and damping behavior. These findings offer a comprehensive understanding of dGTR’s performance and recyclability, informing its integration into sustainable manufacturing efforts, including eco-friendly tires, rubberized asphalt, vibration-dampening components, and energy-efficient building materials. By leveraging advanced thermal analysis, this research contributes to circular economy initiatives and supports the development of high-value applications for recycled rubber.

### 7.7. Sustainable Polymer Composites Based on Hemp Hurd and Recycled Polyolefins

Arun Ghosh *
Troy University
* Correspondence: aghosh@troy.edu

**Abstract:** The growing interest in manufacturing sustainable materials from recycled and bio-renewable feedstock is driven by environmental concerns. This study utilized post-consumer single-use medical personal protective equipment (PPE) fabrics as recycled feedstock. The materials consisted of approximately 80% polypropylene yarns and 20% polyethylene films. Additionally, hemp hurd (HH), a lignocellulosic biomass derived from the stems of industrial hemp, was explored. Industrial hemp is a non-psychoactive variety of Cannabis sativa that is recognized for its effectiveness in carbon sequestration, due to its high rate of CO_2_ absorption and ability to store carbon in both its biomass and the products derived from it [1]. In this study, the HH flakes were ground into fine particles (The material, composed of a 50/50 blend of hemp hurd and PPE, displayed an average tensile strength of 11.4 MPa and a Young’s modulus of 2.8 GPa. When a portion of the PPE phase in the blend was replaced with PPgMA, there was a notable enhancement in both tensile strength and stiffness. Specifically, the blend formulated with HH, PPE, and PPgMA in a ratio of 50/45/5 exhibited a significant increase in mechanical performance, achieving an average tensile strength of 26.5 MPa and a Young’s modulus of 3.2 GPa. Rheological studies indicated that the materials’ storage modulus and complex viscosity were dependent on frequency. As the angular frequency increased, the viscosity decreased while the modulus increased, suggesting that the composites behaved like traditional thermoplastics. Additionally, SEM imaging showed that PPgMA improved interfacial adhesion within the blend, which correlated with enhancements in tensile strength, modulus, and melt viscosity. In summary, bio-renewable hemp hurd has the potential to produce materials with improved mechanical performance and good thermal recyclability, suitable for various technical applications [2].

**References**

1. Muttil, N.; Sadath, S.; Coughlan, D.; Paresi, P.; Singh, S.K. Hemp as A Sustainable Carbon Negative Plant: A Review of Its Properties, Applications, Challenges and Future Directions. *Int. J. Integr. Eng.* **2024**, *16*, 1–12.
2. Ghosh A. Hemp Hurd/Medical PPE Composites: Enhancing Mechanical and Thermoplastic Characteristics. *Polym. Compos*. **2025**, 1–15. https://doi.org/10.1002/pc.70080.

### 7.8. A Sustainable Structural Adhesive and Its Improved Corrosion Resistance in Adhesively Bonded Metal Joints

Amit Naskar * and Zeyang Yu
Oak Ridge National Laboratory
* Correspondence: naskarak@ornl.gov

**Abstract:** Adhesive-bonded joints are essential in multi-material structures for lightweighting and energy efficiency in the automotive industry. The development of robust, repairable adhesives that can also enhance corrosion resistance of the bonded joints is important for both operational feasibility and economic advantages. We developed a lignin-based thermoplastic adhesive that delivers equivalent adhesion strength as that of conventional epoxy-based adhesives (lap shear strength of 20–23 MPa). It demonstrates superior durability and recyclability, maintaining joint strength after prolonged ambient storage and multiple debonding-rejoining cycles. Additionally, it shows enhanced corrosion resistance, with only a 24% lap shear strength reduction after 500 h in corrosive conditions, versus 61% for epoxy adhesives.

## 8. Session: Energetics and Thermal Hazards

### 8.1. Leveraging Controlled-Atmosphere Preparation Methods with Heat Flow Calorimetry to Study Environmental Factors in Explosive Compatibility

John Rosener *, Elizabeth Glascoe, Cody Cockreham and Steven Hawks
Lawrence Livermore National Laboratory
* Correspondence: rosener1@llnl.gov

**Abstract:** Standard chemical compatibility techniques for high explosives are limited in their ability to provide key information on chemical decomposition. Current gas-based methods require specific gas environments, such as vacuum or inert gas, neither of which allows for the study of altered gas environments such as humid air. Microcalorimetric techniques measure heat flow against a reference, which allows for testing in various environments. Differential scanning calorimetry (DSC) is commonly used as an alternative to gas-based test methods; however, fast-heating rates coupled with massive heat release during full decomposition of explosive materials incur challenges in experimental design and interpretation of aging-related chemistry. Heat flow calorimetry (HFC), an isothermal calorimetric technique, offers a uniquely well-suited alternative for the study of explosives compatibility, especially in the study of environmental influence due to its high sensitivity and versatile experimental design.

Here we report on an experimental study of N, N, N′, N′-Tetrakis (2-hydroxypropyl) ethylene-diamine) (aka Quadrol^®^), which is a common additive in commercial adhesives and foams, with several different Nitrate Ester reactions. Characterization methods include heat flow calorimetry in the TAMIII, outgassing investigations, and DSC. Isothermal heating experiments of pure Nitrate Ester-based energetics or Quadrol^®^ proved to be stable with little or no heat release. The mixtures, however, produced significant heat, indicating chemical attack on the energetic. Experiments with controlled gas environment (oxygen and moisture) demonstrated that the reaction was significantly more exothermic in the presence of oxygen; moisture played little or no role in the heat release or chemical behavior.

This study highlights the influence of environmental factors when assessing chemical compatibility of energetics. This is not the first study to explore various atmospheres with explosive or propellant compatibility and stability; rather, these results support a growing body of data that highlights this relatively understudied variable.

This work was performed under the auspices of the U.S. Department of Energy by Lawrence Livermore National Laboratory under Contract DE-AC52-07NA27344. LLNL-ABS-2004136.

### 8.2. Determination of Heat Transfer in Virtual Packaging Systems (VPS) for Cookoff and Self-Accelerating Decomposition Temperature (SADT) Evaluation

Bertrand Roduit *, Patrick Folly, Alexandre Sarbach and Richard Baltensperger
AKTS SA
* Correspondence: b.roduit@akts.com

**Abstract:** Maintaining the quality of temperature-sensitive products is crucial in modern logistics. This requires accurate assessment of both temperature dynamics and kinetic parameters to predict the thermal behavior of materials, such as shelf life and decomposition (aging) rate, under varying temperature conditions. Typically, collected temperature data reflects the ambient or external temperature of the packaging. However, predicting material degradation accurately demands knowledge of internal package temperatures. Two common methods for obtaining this data include placing temperature sensors inside the package or employing a Virtual Packaging System (VPS), which estimates internal temperatures based on external readings. VPS provides an advanced solution by using thermal modelling to optimize conditions across different packaging types.

Measuring the thermal conductivity of materials, particularly in complex, multilayer packaging systems, remains challenging. Existing methods can produce widely varying results. For instance, the hot-disc technique involves placing a thin sensor between layers of material, applying a thermal pulse, and measuring the resulting temperature response. However, such setups often fail to replicate the complexity of real-world, multi-layered systems.

We propose a novel method using temperature sensors, such as T-dataloggers, placed both inside and on the surface of a sample container. By analysing temperature gradients across a known material thickness, caused by external temperature fluctuations, we utilize naturally occurring transient heat sources influenced by environmental conditions. Combined with Fourier’s law, this approach allows for the calculation of thermal conductivity based solely on observed daily temperature variations. This method has previously been applied by us to materials where heat generation during decomposition is minimal, such as vaccines or powdered foods.

In this study, the same approach is extended to high-energy materials, whose heat production at typical ambient temperatures is also often negligible. This allows thermal conductivity to be determined without accounting for decomposition heat, relying solely on temperature fluctuations. By monitoring internal and external temperatures and numerically adjusting the thermal conductivity, it becomes possible to simulate the material’s internal thermal dynamics without the need for hot-disc measurements. Once the thermal conductivity is established, it can be combined with kinetic parameters to predict reaction rates for various sample masses and environmental temperature profiles. These profiles may include conditions typical of SADT studies, or even higher temperatures relevant to slow cookoff ignition scenarios for energetic materials. This innovative approach enables more accurate determination of thermal conductivity and expands the ability to predict the thermal behavior of high-energy materials across different mass scales, packaging types, and temperature conditions.

### 8.3. Nuclear Recriticality Test Study of PCI Based on ASTM E521 & E261 & E228

Yan Peng *
China Institute of Atomic Energy
* Correspondence: pengyan@cnncmail.cn

This paper is focused on the re-criticality study during the Pellet to Cladding Interaction (PCI) phenomenon of a Nuclear Power Plant. During PCI, the criticality instability exists in this intermediate region of density between gaseous and normal densities. As the core expands for uranium and plutonium metal systems in this intermediate density region, holding both the reflector thickness and the fuel mass constant, the volume coefficient of expansion has positive feedback, contrary to the general intuition that an expanding system has a negative coefficient of expansion. An expanding core with fixed core mass becomes more reactive instead of less reactive. The expected behavior is for reactivity to decrease as temperatures rise, and this intermediate density region shows an increase in reactivity as the core expands. The coefficient of expansion is only positive in this intermediate density region. This means that if the inner radius of the reflector changed when the core region was being varied, the outer radius of the reflector changed by the same amount. The basic function of the reflector is to return neutrons to the core where they can cause fission and thus reduce the amount of fissile material required to maintain or achieve criticality. This type of behavior in the intermediate density region is known as the unstable criticality region. So, by “stable” in the criticality sense, it is meant that should the core expand for any reason, the state of criticality will decrease; i.e., the system becomes less critical. By “unstable,” it is meant that the core should expand due to a temperature rise or any other reason, the state of criticality will increase, the existing fission power will rise, and the consequence is unknown. Hence, the reactivity would increase and further increase fission power with the core expanding [1–3].

In this paper, according to the standard of ASTM E521 [4] Standard Practice for Investigating the Effects of Neutron Radiation Damage Using Charged-Particle Irradiation, E261 [5] Standard Practice for Determining Neutron Fluence, Fluence Rate, and Spectra by Radioactivation Techniques and E228 [6] Standard Test Method for Linear Thermal Expansion of Solid Materials With a Push-Rod Dilatometer, the swelling volume of fuel specimen [7] could be measured and calculated under irradiation conditions. Based on the geometry determination of the fuel relocation in PCI, the geometric coefficient Bg [8] and material buckling coefficient Bm [9,10] of fuel shall be known by corresponding neutron fluences, reaction rates, and their physical characteristics. Then, the re-criticality status could be decided by the ratio value of this geometric buckling coefficient Bg to the material buckling coefficient Bm. When this ratio is equal to one, the re-criticality occurs.

Keywords: PCI, re-criticality, geometric buckling, material buckling, ASTM E521& E261& E228

**References**

1. Lewis, W.B. A Practical Approach to Nuclear Criticality Safety II—Critique of a Model. *Nucl. Technol.* **1971**, 12, 276–280.
2. Bandini, B.R.; Baratta, A.J. Potential for Recriticality of the Relocated Core. *Nucl. Technol.* **1989**, *87*, 926–931.
3. Finfrock, S.H. CritView User’s Guide. N-UG-G-00002. Rev. 1. 2019.
4. *ASTM E521*; Standard Practice for Investigating the Effects of Neutron Radiation Damage Using Charged-Particle Irradiation. Annual Book of ASTM Standards; ASTM: West Conshohocken, PL, USA, 2014; Volume 12.02.
5. *ASTM E261*; Standard Practice for Determining Neutron Fluence, Fluence Rate, and Spectra by Radioactivation Techniques. Annual Book of ASTM Standards; ASTM: West Conshohocken, PL, USA, 2014; Volume 12.02.
6. *ASTM E228*; Standard Test Method for Linear Thermal Expansion of Solid Materials with a Push-Rod Dilatometer. Annual Book of ASTM Standards; ASTM: West Conshohocken, PL, USA, 2017; Volume 14.05.
7. *EPRI NP-196*; Research Project 348. Clean Critical Experiment Benchmarks for Plutonium Recycle in LWR’s (Foil Activation Studies). Technical Report; Pacific Northwest National Laboratory (PNNL): Richland, WA, USA, 1978; Volume 2.
8. Itagaki, M.; Miyoshi, Y.; Hirose, H. A Geometric Buckling Expression for Regular Polygons: II. Analyses Based on the Multiple Reciprocity Boundary Element Method. *Nucl. Technol.* **1993**, *103*, 92–402.
9. Rutledge, G.P.; Dobbe, F.A.; Price, C.H. Critical Dimensions of Water Reflected Systems Containing 235U-H2O-Zr. *Nucl. Technol.* **1966**, *2*, 461–467.
10. Bowen, D.G.; Busch, R.D. *Hand Calculation Methods for Nuclear Criticality Safety*; ORNL/TM-2022/2747; Oak Ridge National Laboratory (ORNL): Oak Ridge, TN, USA, 2023.

### 8.4. Explosive Testing and Assessment of In-House Material Standards

Shanti Singh *, Hope Dettweiler, Isa Mohammed and Ricardo Pontes
Canadian Explosives Research Laboratory
* Correspondence: shanti.singh@canada.ca

**Abstract:** Friction and impact sensitivity, in addition to exothermic decomposition energy (−∆H) and onset temperature (T_onset_) of decomposition, are frequently used to characterize and classify energetic materials by United Nations (UN) tests. Test Series 3(aii) and 3(bi) in the UN Manual of Tests and Criteria describe the use of friction and impact test machines to measure the sensitivity of a material to impact and friction stimuli. Results permit classification of the material and determination of whether the material is too dangerous to transport in the form tested. Similarly, differential scanning calorimetry (DSC) is listed in Appendix 6 of the UN Manual as the screening test to measure −∆H and T_onset_ of decomposition of a chemical. DSC data help determine whether a chemical can be excluded from being classified as a Class 1 (Explosive) material, provided that certain threshold values for −∆H and T_onset_ are not exceeded. Two explosives, pentaerythritol tetranitrate (PETN) and cyclonite (RDX), are currently used as in-house standards for assessing the functionality of these sensitivity and thermal test methods. Before the current in-house batches of PETN and RDX are depleted, new batches of each need to be assessed as a replacement to verify consistency. Differences and similarities in the materials and the instrument response are discussed.

© His Majesty the King in Right of Canada, as represented by the Minister of Natural Resources, 2025

## 9. Session: Fast Scan Techniques/Nanocalorimetry

### 9.1. The Rigid Amorphous Fraction in Commercial Isotactic Polypropylene Fibers Using Flash DSC

Yung P. Koh * and Sindee Simon
North Carolina State University
* Correspondence: ykoh@ncsu.edu

**Abstract:** The effects of thermal history on the rigid fraction (rigid amorphous fraction, XR) of commercial isotactic polypropylene (iPP) fibers from Fiber Vision were investigated using Flash DSC. Flash DSC allows the suppression of reorganization during scanning due to the fast crystallization kinetics of iPP through fast scanning rates, resulting in accurate measurement of crystal fraction (XC). The fast-scanning rates of Flash DSC also allow the accurate measurement of both ΔCp at given thermal treatments and ΔCp of 100% amorphous iPP (ΔCpo), resulting in an accurate ΔCp ratio, mobile amorphous fraction XM (XM = ΔCp/ΔCpo). Consequently, Flash DSC enables us to estimate the accurate XR of iPP, obtained indirectly from XC and XM (XR = 1 − XC − XM).

The thermal history includes no treatment (as-received), melt-cooling at broad rates ranging from 3000 to 0.1 K/s (180,000 to 6 K/min), and isothermal hold at 126 °C for times ranging from 0 to 30,000 s after complete amorphization. In melt-cooling conditions, the rigid fraction increases with cooling rate and goes through a maximum at a cooling rate of 500 K/s, while the crystal fraction decreases with cooling rate. In isothermal conditions, the rigid fraction increases with hold time, similar to a higher cooling rate before maximum, but the rigid fraction is constant at longer hold times, while the crystal fraction slightly increases. The simple thermal history of cooling and isothermal hold is found to change significantly the rigid fraction as well as the ratio to crystal fraction (crystallinity), which directly affects the properties of the product in use.

### 9.2. Advancing Crystallization Modeling in Thermoplastic Composites: Incorporating Fiber Effects

Alicyn Rhoades *, Xiaoshi Zhang, Benson Jacob, Richard Schaake, Gijs Kort and Ralph Colby
Penn State—Behrend
* Correspondence: amh234@psu.edu

**Abstract:** Crystallization is a key step in the solidification process during the thermal processing of thermoplastics. This stage significantly influences the dimensional stability and mechanical properties of the final part. Traditionally, the crystallization of thermoplastic composites has been assumed to occur under conditions of slow cooling and high temperatures—scenarios that do not accurately represent real-world processing conditions. Additionally, the influence of fibers is often overlooked. In this talk, we will present our latest advancements in developing a modified Hoffman-Lauritzen model that accounts for the effects of fibers, rapid cooling, and low temperature conditions.

**References**

1. Zhang, X.; Alexander, J.D.; Seo, J.; Gohn, A.M.; Behary, M.J.; Schaake, R.P.; Colby, R.H.; Rhoades, A.M. Crystallization Kinetics of Glass Fiber Filled Poly(Ether Ether Ketone) with Nanogram Sample Size: Feasibility Study for Fast Scanning Calorimetry. *Thermochim. Acta* **2023**, *721*, 179442. https://doi.org/10.1016/j.tca.2023.179442
2. Zhang, X.; Alexander, J.D.; Seo, J.; Jacob, B.J.; Kort, G.D.; Schaake, R.P.; Weigand, S.J.; Rhoades, A.M.; Colby, R.H. Role of Glass Fiber in the Flow-Induced Crystallization of Poly (ether ether ketone). *Macromolecules* **2024**, *57*, 8012–8024.

### 9.3. Tunable Dynamic Fragility and Salt-Enhanced Glass Transition Definition in Polyzwitterion Systems

John Thomas *, Sophia Dinn, Ashleigh Herrera, Ayse Asatekin, Xiaoshi Zhang, Alicyn Rhoades and Peggy Cebe
Tufts University
* Correspondence: john.thomas@tufts.edu

**Abstract:** Polyzwitterions based on the sufobetaine moiety are strong glass formers with high glass transition temperatures [1]. Here we present a systematic study of the changes in the thermodynamic and structural properties of poly(sulfobetaine acrylate), PSBA, which can be tuned by the addition of choline chloride, ChCl. ChCl salt was dissolved in 1:1 H2O:TFE with PSBA in varying molar ratios of monomeric unit to salt ion, SBA:ChCl (1:0, 1:0.125, 1:0.25, and 1:0.5). In prior work we observed that the inclusion of small molecule salts, such as LiCl, resulted in the disruption of dipolar crosslinking, reducing the overall rigidity of the polymer network [2]. Addition of ChCl to PSBA allows us to compare the effects of a cation much larger than Li^+^ while maintaining the same Cl^−^ anion. Using Thermogravimetric Analysis, we observe that a reduction in sidechain rigidity leads to a reduction in the onset of thermal degradation. Temperature Modulated Differential Scanning Calorimetry was used to determine both the specific reversing heat capacity and the glass transition temperature, Tg, as a function of ChCl content. The inclusion of ChCl causes disruption of electrostatic crosslinking and plasticizes PSBA, systematically reducing Tg from a maximum of 182 °C in homopolymer PSBA to 108 °C in the 1:0.5 PSBA/ChCl sample. Using Fast Scanning Calorimetry, the dynamic fragility of PSBA/ChCl was measured at a constant heating rate of 2000 K s^−1^ while the cooling rate varied systematically from 4000 K s^−1^ to 300 K s^−1^ at 100 K s^−1^ intervals. This relatively fine-grained rate sampling results in an improvement in the calculated fictive temperatures, Tf, and we observe less noise in Tf as ChCl content increases. A bootstrap technique was employed to predict the spread in the expected fictive temperatures for each sample, which were then fit using the Williams-Landell Ferry equation to obtain the fragility indices, m. The homopolymer showed the highest fragility index m = 103, while the 1:0.125 sample showed the lowest fragility index m = 57. As salt content increases, the fragility increases but does not recover the original homopolymer value, with the 1:0.5 sample having a fragility index m = 69. These result shows that the dynamic fragility can be tuned through salt addition, which allows for tailoring both the packing efficiency and the overall rigidity of the polymer/salt complex.

Research funded by NSF DMR-2003629.

**References**

1. Clark; Biswas, Y.; Taylor, M.E.; Asatekin, A.; Panzer, M.J.; Schick, C.; Cebe, P. Glass-Forming Ability of Polyzwitterions. *Macromolecules* **2021**, *54*, 10126–10134.
2. Thomas; Chum, S.; Deucher, W.; Mondal, A.; Asatekin, A.; Cebe, P., Thermal and structural properties of polyzwitterions: Effects of monomer chemistry and salt addition. *Thermochim. Acta* **2023**, *730*, 179617.

### 9.4. Rapid Thermal Decomposition of Viton-A

Mirko Schoenitz * and Edward L Dreizin
New Jersey Institute of Technology
* Correspondence: schoenit@njit.edu

**Abstract:** Viton-A, when used as a binder in energetic formulations, typically decomposes on millisecond time scales. Characterization of this reaction, however, has been traditionally carried out using conventional thermal analysis, and on time scales of minutes to hours. Here, the decomposition of thin films of Viton-A is studied using fast scanning calorimetry with heating rates up to 20,000 K/s and in an inert atmosphere. The measurements are complemented by visual observation of the sensor, allowing for correlating details of the heat flow signal with processes occurring at particular parts of the sensor. As in conventional TA in inert environments, the heat flow signal does not return to an identifiable baseline, making interpretation of the results challenging. Nevertheless, it is observed that the volatilization is accompanied by an exothermic heat effect that slightly precedes the removal of the material, or mass loss in conventional thermal analysis. Kinetic processing shows an activation energy of 218 ± 2 kJ/mol, consistent with literature data.

### 9.5. Impact of Lamellar Infill and Thickening on the Rigid Fraction of Semicrystalline PLLA

Logan Williams * and Sindee Simon
North Carolina State University
* Correspondence: lwilli25@ncsu.edu

**Abstract:** Poly(L-lactic acid) (PLLA) is a well-known semicrystalline polymer with three distinct phases: the crystalline, mobile amorphous, and rigid phases. The rigid phase (or rigid fraction (RF)) is thought to be the layer of polymer chains with properties between those of the crystal phase and mobile amorphous phase, such as restricted mobility. Fast differential scanning calorimetry (Flash DSC) is used to determine the relationship between primary crystallization and the rigid fraction of PLLA, as well as the relationship between secondary crystallization and the rigid fraction. The rigid fraction shows a positive trend with primary crystallization, regardless of the temperature of crystallization. Lamellar infill, achieved by crystallizing at one temperature followed by crystallization at a lower temperature, results in an increased rigid fraction content at the same degree of crystallinity relative to primary crystallization alone. Ultra-long crystallization experiments at a chosen temperature reveal a sudden increase in the melting temperature, consistent with lamellar thickening. The relationship between lamellar thickening, lamellar infill, and the rigid fraction is examined.

### 9.6. Crystallization of Polyamide 66 Blends

Xiaoshi Zhang *, John Buzinkai, René Androsch, Ralph Colby and Alicyn Rhoades
Penn State—Behrend
* Correspondence: xvz5402@psu.edu

**Abstract:** Many polymers exhibit bimodal, temperature-dependent crystallization kinetics, characterized by two distinct crystallization time minima at low and high temperatures. These regimes are typically governed by different nucleation mechanisms and are often associated with the formation of different crystal polymorphs. In this work, we focus on PA66 and its blends under conditions involving fast cooling and high undercooling. To investigate crystallization behavior under such conditions, Flash Scanning Chip Calorimetry (FSC) was employed to study the crystallization kinetics of PA66 and its blends with poly(hexamethylene isophthalamide-co-terephthalamide) (PA6I/6T) and polycaprolactam (PA6). These blends offer a unique opportunity to systematically modulate the crystallization kinetics of PA66 across both nucleation regimes. For the PA66/PA6I/6T blends, our results show that the selection between mesophase and α-phase is governed by a temperature-dependent switch in nucleation mechanism from heterogeneous to homogeneous nucleation. This observation rules out purely kinetic control as the primary cause of polymorph selection under deep undercooling. For the PA66/PA6 blends, we find that the transition between the two nucleation mechanisms can be altered, an effect not observed in the homopolymers. Detailed thermal, spectroscopic, and morphological characterizations are presented to support these findings.

## 10. Session: Electronic Packaging Materials

### 10.1. Advanced Metrology Suite for Linking Residual Stress to Fundamental Properties of Thermoset Packaging Materials

Stian Romberg *, Polette Centella, Ran Tao, Alexander Landauer, Karl Schoch, Huong Giang Nguyen, Gale Holmes, Gery Stafford and Christopher Soles
National Institute of Standards and Technology
* Correspondence: s.k.romberg@gmail.com

**Abstract:** Highly filled thermoset composites are frequently used to encapsulate and protect components in semiconductor packages. Cure-induced shrinkage and changes in the ambient hygrothermal state generate residual stresses between thermoset composites and the inorganic components they surround. These residual stresses can lead to warpage and delamination, limiting the package yield and lifetime. Here, we present a suite of metrologies and methodologies to demonstrate how fundamental measurements foretell the evolution of residual stress in a commercial “glob-top” encapsulant. Differential scanning calorimetry (DSC) reveals complex kinetics with overlapping reaction peaks that can be described using advanced modeling. Dilatometry conducted using a custom digital image correlation setup is used to characterize the magnitude and evolution of the cure shrinkage. Traditional and advanced rheological techniques identify two gelation-like phenomena, providing predictions for when cure shrinkage should begin to manifest as residual stress. Finally, two distinct residual stress measurements are conducted by coating silicon wafers with a thin layer of the glob-top encapsulant. The fundamental DSC, dilatometry, and rheometry measurements provide insight into the evolution of residual stress that we observe during curing and under thermal cycling after curing. These results emphasize the importance of fundamental metrologies in predicting the evolution of residual stress in thermoset composites used in semiconductor packaging.

### 10.2. Thermal Interface Materials for Advanced Packaging: The Role of Thixotropic Rheology

Ran Tao ^1,^*, Stian Romberg ^1^, Amanda Forster ^1^, Christopher Soles ^1^, Kaitlyn Hartog ^1^ and Karl Schoch ^2^
^1^ National Institute of Standards and Technology
^2^ Northrop Grumman
* Correspondence: ran.tao@nist.gov

**Abstract:** Thermal interface materials (TIMs) are essential components in advanced packaging, enabling efficient heat dissipation from chips and through the package structure. They play a critical role in thermal management, which has become one of the primary bottlenecks in high-performance electronic systems. Rheological properties are key to optimizing manufacturing and assembly processes. In this work, we study the linear viscoelastic regime and thixotropic behavior (shear thinning and recovery) of an aluminum nitride (AlN)-filled, two-part, cure-in-place silicone gap filler and report its implications for assembly processes, including dispensability and retention after deposition. These rheological characteristics are crucial for ensuring gap-filling accuracy and optimizing bondline thickness, as well as for avoiding “pump out” (flow into keep-out zones) during final assembly. However, this paste-like behavior masks the most common indicator of the gel point (the crossover of the storage and loss modulus). Further, the material exhibits a limited linear viscoelastic regime, preventing the use of alternative gel point measurements that depend on traditional multi-frequency measurement approaches that require long acquisition periods or apply large strains. To address these issues, we implement an advanced multi-frequency rheology technique, namely optimally windowed chirps (OWCh), to determine the gel point during curing.

### 10.3. Experimental Investigation and Modeling of Direct Immersion Cooling for Electronics Thermal Management

Hrushikesh Jadhav *, Runshuang Guo and Solomon Adera
University of Michigan
* Correspondence: kalakar@umich.edu

**Abstract:** The push toward vehicle electrification has amplified the need for advanced thermal management solutions that can efficiently remove highly localized heating. Recent advances in vehicle electrification employ wide band gap semiconductors (WBG) such as silicon carbide (SiC) and gallium nitride (GaN) power transistors and high electron mobility transistors (HEMT) for on-board and fast charging. To take advantage of their extraordinarily fast switching capabilities, WBG semiconductors are also used in the power train, inverters, and converters. These devices, however, generate highly localized heating that exceeds 1 kW/cm^2^ during operation. Such hotspots are challenging to cool using conventional thermal management approaches. This study presents an experimental and numerical investigation of direct immersion cooling as a viable thermal management solution for SiC field-effect transistors (FET), with direct implications for battery pack cooling in high-power EV architectures. The emerging direct immersion cooling is attractive since it eliminates intermediate thermal resistances associated with heat spreaders and thermal interface materials by submerging the live device directly in a thermally conductive and electrically insulating viscous fluid. The direct contact between the working fluid and the device enables efficient cooling. Compared to traditional air and/or single-phase liquid cooling, our immersion cooling demonstrated a high heat transfer coefficient (HTC), achieving values up to 820 W/m^2^·K along with 400 kW/m^2^ of heat flux dissipation. These results were validated using ANSYS Icepak simulations that showed strong agreement with measured temperatures (±2 °C), which were measured using a four-wire Kelvin setup. By demonstrating the effectiveness of immersion cooling on SiC FET, critical components in powertrain and fast-charging units, this study offers a foundational framework for applying the same thermal approach to densely packed lithium-ion battery modules. With the rising heat fluxes during fast charging and discharging cycles, especially under high C-rate conditions, immersion-based cooling techniques hold promise in improving battery pack longevity, reducing thermal runaway risks, and enabling compact, high-performance electric vehicle thermal designs. The insights gained from this study have direct implications in the co-optimization of power electronics and battery cooling, a necessary step for advancing next-generation electric mobility systems.

### 10.4. Measurement of Cure Stress During Cure of Thermosets and Subsequent Thermal Cycling

Karl Schoch *, Kortney Kersten and Philip Panackal
Northrop Grumman
* Correspondence: k.eric.schoch@ngc.com

**Abstract:** Thermosetting materials shrink when they cure, which can damage parts that are coated or encapsulated with those materials. Examples include cracking, delamination, warping, and void formation. Many different measurements have been used to measure shrinkage and the resulting residual stress remaining in coated or encapsulated parts. Direct measurement of shrinkage by thermomechanical analysis, dynamic mechanical analysis, dilatometry, and changes in density have been reported. In addition, there is published work on the measurement of residual stress using cantilever bending and warping by interferometry.

In the present work, we coated and polished silicon wafers with commercial encapsulants and measured the radius of curvature during cure and with thermal cycling after cure. In this paper, those test results are compared to the determination of changes in shear modulus, film stress, density, and shrinkage during the cure of the same materials. All of the materials were heat-cured, filled epoxy formulations. There was a transient increase in film stress associated with the gel point from the shear modulus measurement.

Film stress test results have also been compared to properties of the cured material during cycling. All of the materials had minimal stress in the cured condition at the cure temperature. While cooling from the cure temperature, the stress increased after the material passed through its glass transition. With subsequent thermal cycling, the residual stress decreased as the material was heated and reached a zero-stress condition as it passed through the glass transition region. With repeated thermal cycling, in some cases, the cure advanced slightly, as indicated by an increase in the zero-stress temperature. The residual stress at room temperature after cure could be as high as 20 to 30 MPa and did not decrease with repeated thermal cycling.

## 11. Posters

### 11.1. Oscillatory Rheological Analysis of Poly(Vinylidene Fluoride) Solutions Made from γ-Valerolactone

Anuja Jayasekara *, Peggy Cebe, Lily Buyea and Karin Wyatt
Tufts University
* Correspondence: anuja.jayasekara@tufts.edu

**Abstract:** Poly(vinylidene fluoride) (PVDF) is a semi-crystalline polymorphic polymer utilized in a wide range of applications, including sensors, coatings, and filtration devices [1,2]. The processing of PVDF often involves hazardous solvents such as N,N-dimethylacetamide (DMAc), N,N-dimethylformamide (DMF), and N-methyl-2-pyrrolidone (NMP) [3,4]. These solvents present various hazards, including reprotoxicity, pulmonary toxicity, flammability, and corrosiveness. Therefore, it is essential to explore safe alternative solvents for processing PVDF. &gamma;-valerolactone (GVL) is an eco-friendly, non-hazardous solvent for PVDF processing [5]. However, the processing of PVDF using GVL involves dissolving it at elevated temperatures, such as 100 °C, and when the PVDF/GVL solution cools down, it forms a gel. Therefore, it is crucial to explore the flow properties of the PVDF/GVL solutions for targeted applications in filtration membranes. This study presents a comprehensive rheological analysis of PVDF/GVL solutions using parallel-plate oscillatory shear rheological tests. Control variables include solution concentration, amplitude, frequency, and temperature variation.

Funding Sources: NSF Grant DMR-2003629 and John F. Burlingame Fellowship.

**References**

1. Broadhurst, M.; Davis, G. Physical Basis for Piezoelectricity in PVDF. *Ferroelectrics* **1984**, *60*, 3–13.
2. Govinna, N.; Kaner, P.; Ceasar, D.; Dhungana, A.; Moers, C.; Son, K.; Asatekin, A.; Cebe, P. Electrospun Fiber Membranes from Blends of Poly (Vinylidene Fluoride) with Fouling-Resistant Zwitterionic Copolymers. *Polym. Int.* **2019**, *68*, 231–239.
3. Jayasekara, A.S.; Cebe, P. Quantitative Analysis of Polar Crystalline Fractions in Poly(Vinylidene Fluoride) Electrospun Fibers and Electrosprayed Films. *Polymers* **2023**, *281*, 126140.
4. Jayasekara, A.S.; Mazzaferro, L.; O’Hara, R.; Asatekin, A.; Cebe, P. Hydrophobic Fouling-Resistant Electrospun Nanofiber Membranes from Poly (Vinylidene Fluoride)/Polyampholyte Blends. *Soft Matter.* **2024**, *20*, 8654–8662.
5. Ravikumar, V.R.; Schröder, A.; Köhler, S.; Cetinel, F.A.; Schmitt, M.; Kondrakov, A.; Eberle, F.; Eichler-Haeske, J.-O.; Klein, D.; Schmidt-Hansberg, B. Γ-Valerolactone: An Alternative Solvent for Manufacturing of Lithium-Ion Battery Electrodes. *ACS Appl. Energ. Mater.* **2021**, *4*, 696–703.

### 11.2. Application of Modulated DSC Methods on Cooling for Analysis of Petroleum-Based Products

Jennifer Schott *
TA Instruments—Waters, LLC
* Correspondence: jennifer_schott@waters.com

**Abstract:** Modulated Differential Scanning Calorimetry (DSC) methods are commonly used to aid in the interpretation of challenging thermograms for materials where transitions may be difficult to detect. It has been successfully used to investigate features such as glass transitions and crystalline perfection. In this study, a variety of commonly used petroleum-based personal care ointments were explored using standard and modulated DSC. Different cooling rates were used for both methods. The samples exhibited complex peaks on cooling, and there was a clear variability in the onset with respect to different cooling rates. Conversely, similar peak temperatures were maintained regardless of the rate used. To further probe this, a method referred to as ‘modulated heat only’ was employed. This method can be used to automatically limit the amplitude of the sinusoidal temperature modulation based on the selected heating rate and period. In this case, it was used on the cooling ramp and controlled such that the instantaneous rate did not go above 0 °C/min.

### 11.3. Next-Gen Skincare: Rheological Insights into Silver-Enhanced Aloe Gel vs. Commercial Hand Creams

Tien Nguyen ^1,^*, Brooke Wilcox ^1^ and Isaac Roscoe Finch ^2^
^1^ Central Michigan University
^2^ University of Michigan
* Correspondence: nguye7tn@cmich.edu

**Abstract:** This study investigates the incorporation of silver nanoparticles into Aloe vera gel to enhance its moisturizing properties, with the goal of outperforming commercial hand creams. We conduct rheological measurements—including steady shear, oscillatory frequency sweeps, and temperature sweeps—at 25 °C and 37 °C to evaluate flow behavior and thermal stability. Results are compared across pure Aloe gel, nanoparticle-infused gel, and commercial formulations.

### 11.4. Integration of DSC and ARC Data for Determination of TMRad in DTBP/Toluene Systems with Various Concentrations

Bertrand Roduit *, Patrick Folly, Alexandre Sarbach and Richard Baltensperger
AKTS SA
* Correspondence: b.roduit@akts.com

**Abstract:** Runaway reactions are critical safety concerns in chemical processing and are commonly investigated using Accelerating Rate Calorimetry (ARC), typically operated in isothermal (ISO-ARC) or heat-wait-search (HWS) modes. In this study, we present a combined approach utilizing Differential Scanning Calorimetry (DSC) data in conjunction with ARC measurements to enhance the determination of the Time to Maximum Rate under adiabatic conditions (TMRad).

This methodology was applied to systems containing various concentrations (5–25 wt%) of di-tert-butyl peroxide (DTBP) dissolved in toluene. Kinetic parameters were precisely determined using AKTS-Thermokinetics software, enabling accurate prediction of runaway behavior under true adiabatic conditions (Φ = 1). The dependence of TMRad on initial temperature was evaluated and presented in the form of thermal safety diagrams. Critical initial temperatures corresponding to a TMRad = 24 h were established for each DTBP concentration studied.

To validate the kinetic parameters, data from non-isothermal and isothermal DSC experiments, as well as adiabatic ARC measurements with Φ > 1, were employed. The proposed methodology allows for the optimal selection of initial adiabatic temperatures across a range of concentrations, thereby significantly reducing the time required for adiabatic investigations. This integrated approach provides a practical and efficient strategy for assessing thermal hazards and supports safer design of chemical processes involving reactive mixtures.

